# Functional interplay between the transcription factors USF1 and PDX-1 and protein kinase CK2 in pancreatic β-cells

**DOI:** 10.1038/s41598-017-16590-0

**Published:** 2017-11-27

**Authors:** Sarah Spohrer, Rebecca Groß, Lisa Nalbach, Lisa Schwind, Heike Stumpf, Michael D. Menger, Emmanuel Ampofo, Mathias Montenarh, Claudia Götz

**Affiliations:** 1 Medical Biochemistry and Molecular Biology, Saarland University, Homburg, Germany; 2 Institute for Clinical & Experimental Surgery, Saarland University, Homburg, Germany

## Abstract

Glucose homeostasis is regulated by insulin, which is produced in the β-cells of the pancreas. The synthesis of insulin is controlled by several transcription factors including PDX-1, USF1 and USF2. Both, PDX-1 and USF1 were identified as substrates for protein kinase CK2. Here, we have analysed the interplay of PDX-1, USF1 and CK2 in the regulation of PDX-1 gene transcription. We found that the PDX-1 promoter is dose-dependently transactivated by PDX-1 and transrepressed by USF1. With increasing glucose concentrations the transrepression of the PDX-1 promoter by USF1 is successively abrogated. PDX-1 binding to its own promoter was not influenced by glucose, whereas USF1 binding to the PDX-1 promoter was reduced. The same effect was observed after inhibition of the protein kinase activity by three different inhibitors or by using a phospho-mutant of USF1. Moreover, phosphorylation of USF1 by CK2 seems to strengthen the interaction between USF1 and PDX-1. Thus, CK2 is a negative regulator of the USF1-dependent PDX-1 transcription. Moreover, upon inhibition of CK2 in primary islets, insulin expression as well as insulin secretion were enhanced without affecting the viability of the cells. Therefore, inhibition of CK2 activity may be a promising approach to stimulate insulin production in pancreatic β-cells.

## Introduction

Protein kinase CK2, which is composed of two catalytic α- or α′-subunits and two non-catalytic β-subunits, phosphorylates more than 400 different substrates of the human proteome. Among these substrates are a number of transcription factors whose transactivation factor activity was either enhanced or repressed upon phosphorylation by CK2^1–6^. Recently, the upstream stimulatory factor USF1 has been identified as a new substrate for CK2^7^. Together with the second member of the USF family, namely USF2, both are involved in the transcriptional regulation of various genes whose gene products are implicated in the stress and immune response, cell cycle regulation, DNA repair and proliferation of cells and in lipid and carbohydrate metabolism^8–12^. Only USF1, but not USF2 was phosphorylated by CK2 and the major phosphorylation site was mapped to threonine 100^7^. Transactivation studies revealed that inhibition of the CK2 phosphorylation of USF1 stimulated the transactivation of some promoters such as the glucokinase promoter and the fatty acid synthetase promoter but not of the heme-oxygenase-1 promoter. Moreover, inhibition of the CK2 phosphorylation of USF1 led to an enhanced binding of USF1 to USF2. In another study it was shown that only a nuclear sub-population of CK2α and CK2β proteins bound to USFs^13^. One interpretation of these results might be that binding of CK2 to USFs facilitates phosphorylation of nuclear USF1. Another possibility might be that the USFs target CK2 to other substrates in the transcription factor complex in the nucleus. However, CK2 was not found within the transcription factor complex of USF1/USF2 at the DNA.

Over the last couple of years CK2 was found to regulate another transcription factor, namely PDX-1 which is directly implicated in the regulation of the transcription of the insulin gene in pancreatic β-cells^4,14^. PDX-1 binds to its own promoter^15^ and regulates its expression in an auto-regulatory loop involving USFs bound to the E-box motif within the proximal PDX-1 promoter^16^. Expression of a dominant negative form of USF2 decreased the binding of USFs to the promoter, which resulted in a lower level of PDX-1 mRNA^17^. These various results prompted us to study the interplay of USF1 with protein kinase CK2 and within the regulation of the PDX-1 expression in the rat glucose-sensitive pancreatic β-cells (INS-1).

We found that PDX-1 and USF1 interact functionally at the PDX-1 promoter in INS-1 cells. The interaction of both proteins and the transcriptional activity are influenced by glucose and by the inhibition of CK2 activity. Both treatments abrogate the transrepressing effect of USF1 on the PDX-1 driven transcription of PDX-1. The measurable impact of CK2 inhibition in primary islets was an enhancement of insulin expression and secretion.

## Results

PDX-1 and USF1 are transcription factors deeply involved in the regulation of glucose homeostasis. In addition, PDX-1 is the key transcription factor for the development of the pancreas and for maintaining the integrity of pancreatic β-cells. Both proteins have been described by us as substrates of protein kinase CK2^4,7^. We have now attempted to find out whether there is an influence of the CK2 phosphorylation on the functions of one or both transcription factors. For the experiments described here, we used the glucose-responsive pancreatic β-cell line INS-1 from rat^18^.

**Figure 1:**
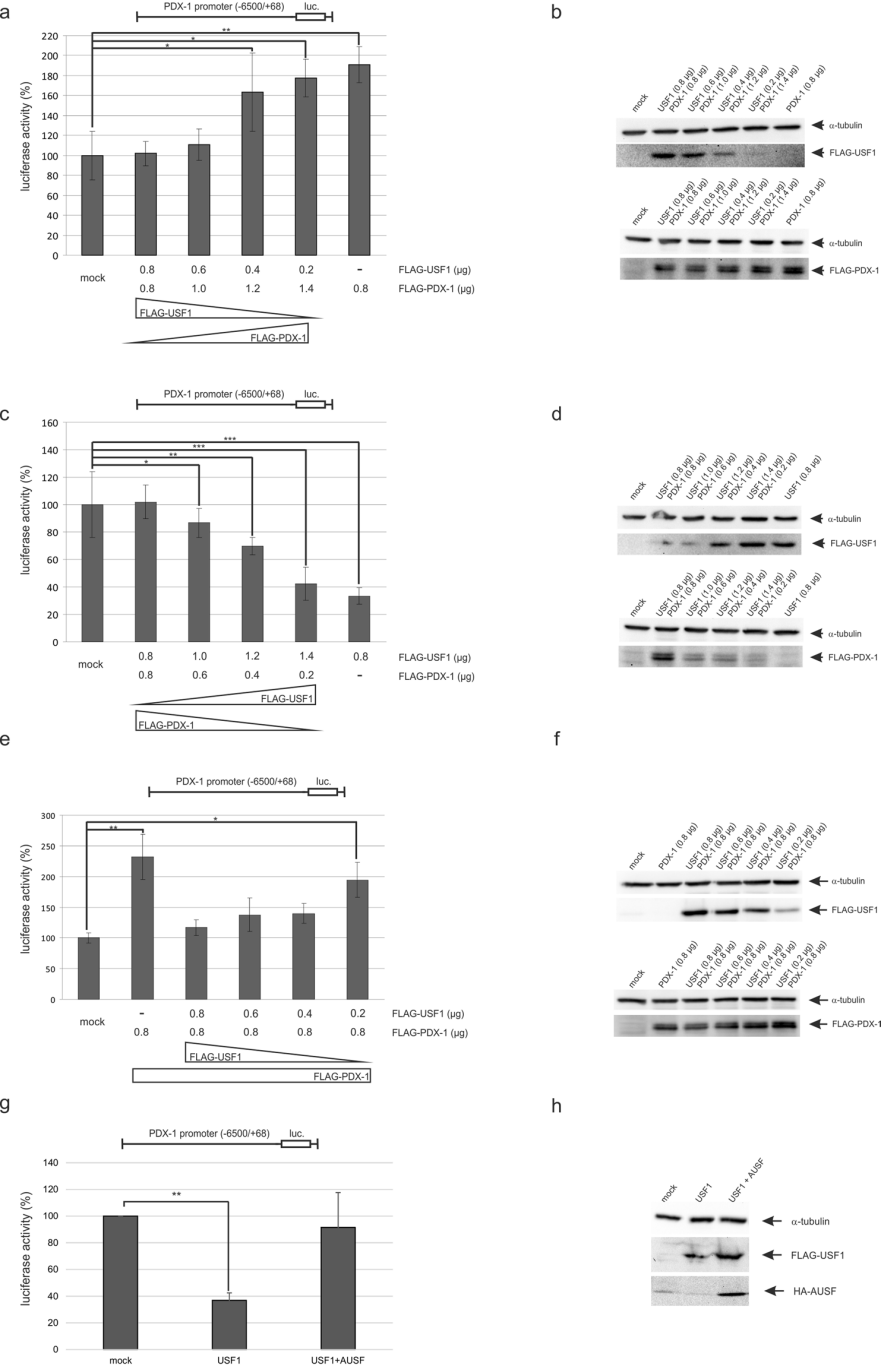
Reporter assays after transfection of INS-1 cells with USF1 and PDX-1 at different concentrations. (**a**) INS-1 cells were transfected with the PDX-1 promoter construct −6500/+68-STF-luc and with different amounts of FLAG-USF1 (0.8 µg–0.2 µg) and FLAG-PDX-1 (0.8 µg–1.4 µg) or the empty vector (mock) as a control. (**c**) INS-1 cells were transfected with the PDX-1 promoter construct −6500/+68-STF-luc and with different concentrations of USF1 (0.8 µg–1.4 µg) and PDX-1 (0.8 µg–0.2 µg) or the empty vector (mock) as a control. Luciferase activity was determined in quadruple; the activity in the mock transfected cells was set to 100%. Statistical analysis was performed by using Students t-test, *Significant difference p < 0.05, **significant difference p < 0.01, ***significant difference p < 0.001. (**b**) and (**d**) The corresponding Western blot analysis of the FLAG-tagged USF1 and the FLAG-tagged PDX-1 is shown aside of each graph. Identification of FLAG-USF1 was performed with the rabbit polyclonal antibody sc-8983, PDX-1 was detected with the polyclonal rabbit antiserum against recombinant full-length mouse PDX-1. Due to the similar molecular weight of USF1 and PDX-1 we performed two Western blots. An α-tubulin antibody was used to demonstrate equal loading of the different samples. (**e**) INS-1 cells were transfected with the PDX-1 promoter construct −6500/+68-STF-luc and with different concentrations of FLAG-USF1 (0.8 µg–0.2 µg) and with 0.8 µg FLAG-PDX-1 or the empty vector (mock) as a control. (**f**) The corresponding Western blot analysis of the FLAG-tagged USF1 and the FLAG-tagged PDX-1 is shown. Identification of FLAG-USF1 was performed with the rabbit polyclonal antibody sc-8983, PDX-1 was detected with the polyclonal rabbit antiserum against recombinant full-length mouse PDX-1. Due to the similar molecular weight of USF1 and PDX-1 we performed two Western blots. An α-tubulin antibody was used to demonstrate equal loading of the different samples. (**g**) INS-1 cells were transfected with the PDX-1 promoter construct −6500/+68-STF-luc and with FLAG-USF1 alone or in combination with the dominant-negative USF-mutant CMV566 A-USF (AUSF) or the empty vector (mock) as a control. (**h**) The corresponding Western blot analysis of the FLAG-tagged USF1 and the HA-tagged AUSF is shown. Identification of FLAG-USF1 was performed with the mouse monoclonal antibody FLAG M2 (F1804), HA-tagged USF with the mouse monoclonal antibody 12CA5. An α-tubulin antibody demonstrates equal loading of the different samples. Full-length blots are presented in Supplementary Figure S2.

Amemiya-Kudo *et al*. reported a functional interplay of USF1 and PDX-1 at the PDX-1 promoter^16^. To investigate whether this interaction also occurs in INS-1 cells under our experimental conditions, we first performed a transactivation assay using a luciferase construct under the control of the complete PDX-1 promoter (−6500/+68). In addition to the reporter construct we also transfected varying amounts of FLAG-USF1 and FLAG-PDX-1 or an empty vector as control (mock). When using more PDX-1 than USF1 the reporter assay showed a transactivation of the PDX-1 promoter construct (180% of the basal activity at a sevenfold excess of PDX-1 over USF1) (Fig. 1a). In contrast, using more of the USF1 construct than of the PDX-1 construct ended up in transrepression of the PDX-1 promoter (40% of the basal activity at a sevenfold excess of USF1 over PDX-1) (Fig. 1c). When performing transfection with a single construct we observed a transactivation with PDX-1 as described in the literature; however, we observed a transrepression with USF1. Figure 1b and d show corresponding Western blot analyses for USF1 and PDX-1 as well as for a loading control. Thus, we conclude that the fate of PDX-1 transcription is dependent on the concentration of both players in the DNA-bound transcription complex. Figure 1c let us conclude that USF1 has a transrepressing effect on the PDX-1 promoter; however, as we varied the amount of both proteins the effect could also be explained by a decreased PDX-1 amount. In a next experiment, we therefore maintained a constant amount of PDX-1 (0.8 µg) and decreased successively the amount of USF1 from 0.8 µg to 0.2 µg (Fig. 1e,f). Figure 1 f shows the Western blot analysis of FLAG-USF1 and FLAG-PDX-1 with respect to the loading control. In a reporter experiment with solely PDX-1 the basal activity of the reporter construct is enhanced by 2.3 fold, meaning that PDX-1 transactivated its own promoter (see also Fig. 1a). When using equal amounts of USF1 and PDX-1 the luciferase reporter activity is repressed to the basal level of mock-transfected cells, as already shown in Fig. 1c. Steadily decreasing the amount of USF1 attenuates the repression; with a fourfold excess of PDX-1 over USF1 we observed a twofold increase of the reporter activity. Thus, we confirmed that USF1 exerts a dose-dependent transrepressing effect on the PDX-1 promoter. To be unequivocally sure that the effect is specific for USF1, we repeated the reporter experiment with USF1 in the presence of a dominant-negative mutant (AUSF)^19^. The AUSF sequence includes an N-terminal HA tag along with the acidic extension and the HLH-B-ZIP region of USF and exerts a dominant-negative effect on the DNA-binding activity of USFs. As shown in Fig. 1g, the overexpression of USF1 led to the repression of the reporter activity. Upon simultaneous transfection of the dominant-negative mutant AUSF the effect is reversed and the reporter activity nearly reached the level of mock-transfected cells. Figure 1h shows the corresponding Western blot analysis. Thus, we could show that the observed effect is obviously USF1-specific.

For the pancreatic β-cell line MIN6 it is known that PDX-1 as well as protein kinase CK2 shuttle from the cytoplasm to the nucleus with increasing concentrations of glucose^20^. Thus, we were first interested in the cellular localization of the transcription factors USF1, USF2 and PDX-1 and protein kinase CK2 (see Supplementary Figure S1). Both subunits of CK2 were present in both compartments with a predominance of the cytoplasmic over the nuclear subpopulation. USF1 and USF2 were both found exclusively in the nucleus, whereas the majority of PDX-1 was found in the nucleus and a minor amount in the cytoplasm as well. We never observed a glucose-dependent change of the localization of PDX-1.

**Figure 2:**
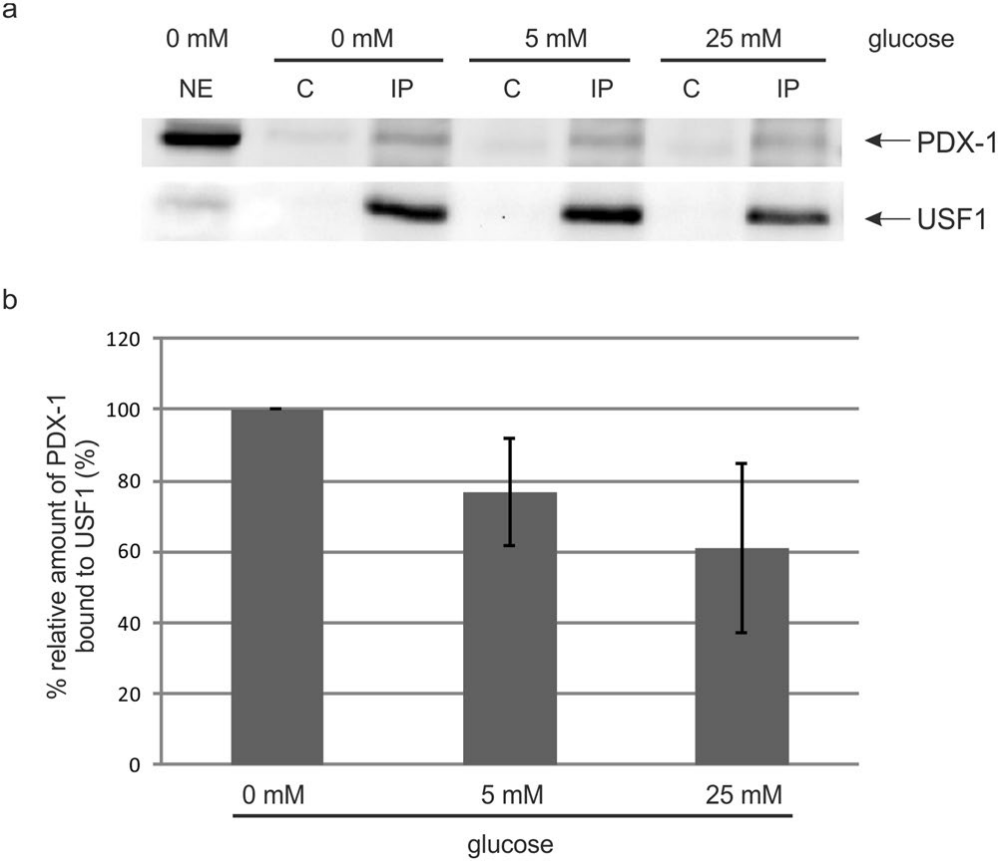
Co-immunoprecipitation of PDX-1 and USF1. (**a**) INS-1 cells were seeded on a 14.5 cm culture plate and starved overnight. The next day, cells were treated with 0 mM, 5 mM or 25 mM glucose and after a period of 4 h cytoplasmic and nuclear proteins were extracted as described in material and methods. Two mg nuclear extract were incubated with the USF1 specific antibody sc-8983 for 2 h, immunocomplexes were loaded on a 10% SDS polyacrylamide gel and transferred onto a PVDF membrane. PDX-1 was identified with the polyclonal rabbit antiserum against recombinant full-length mouse PDX-1, detection of USF1 with the USF1 specific antibody sc-8983 served as a control for immunoprecipitation. NE: 50 μg nuclear extract; C: control precipitate; IP: immunoprecipitate. (**b**) Relative protein amounts of bound PDX-1 to USF1 were shown in the bar graphs. The diagram shows the mean ± SD of three independent experiments. The relative intensities of bound PDX-1 protein to USF1 were normalized to the protein content of precipitated USF1. Full-length blots are presented in Supplementary Figure S2.

A prerequisite for a functional interplay is the physical interaction of the partners. We performed a co-immunoprecipitation analysis of USF1 and PDX-1 from INS-1 cells grown with 0 mM, 5 mM or 25 mM glucose for 4 h. Equal amounts of nuclear extracts were immunoprecipitated with an USF1-specific antibody, precipitated proteins were analysed by Western blot analysis with a PDX-1 as well as an USF1 specific antibody. As demonstrated in Fig. 2a PDX-1 was co-immunoprecipitated with USF1 at every glucose concentration. Figure 2b shows a quantification of the relative amount of PDX-1 bound to USF1 from three independent experiments. With higher glucose concentration less PDX-1 is bound to USF1; however, this change was not statistically significant.

**Figure 3:**
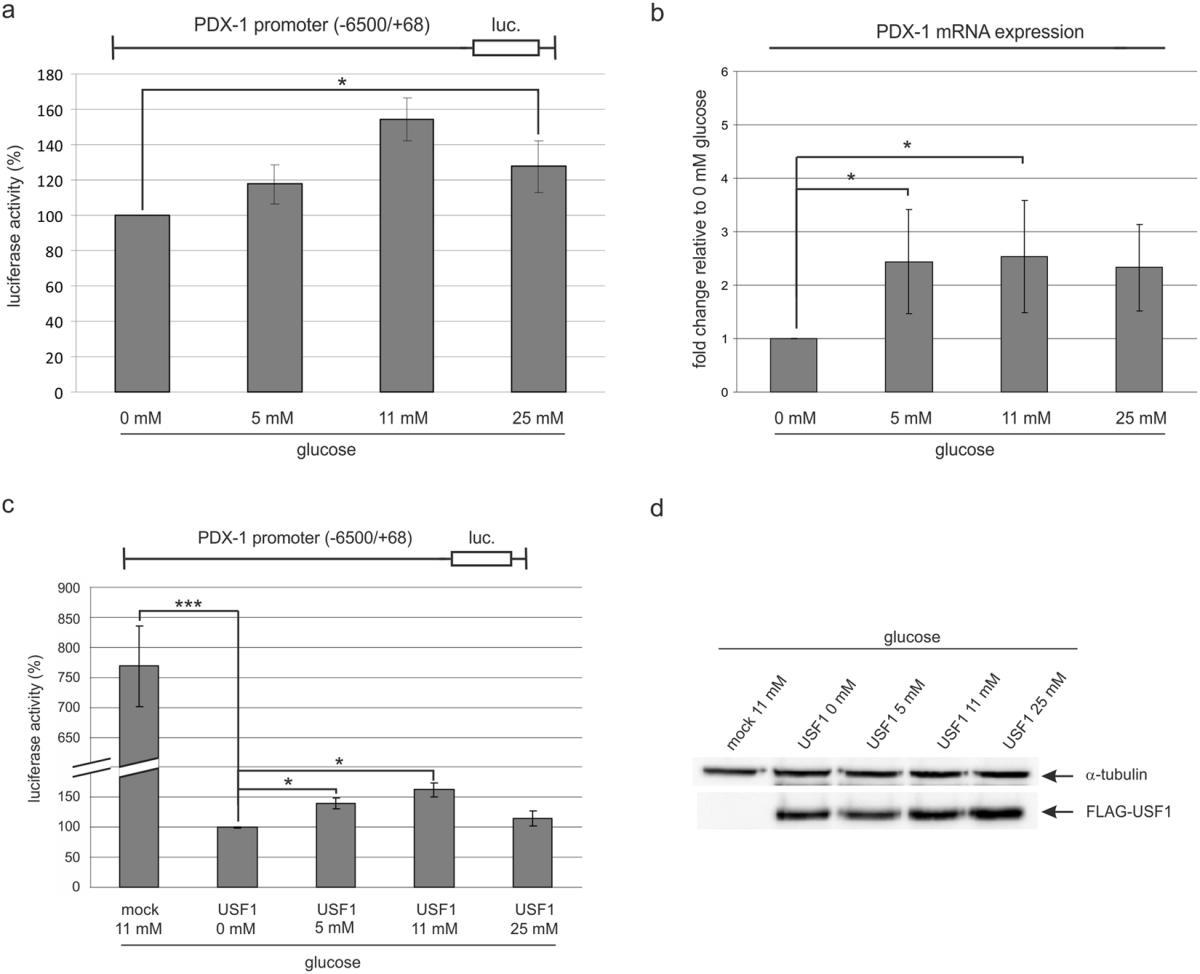
Influence of glucose on the transcription of the PDX-1 promoter of INS-1 cells. (**a**) INS-1 cells were transfected with the PDX-1 promoter construct −6500/+68-STF-luc and starved overnight. The next day, cells were treated with 0 mM, 5 mM, 11 mM or 25 mM glucose and harvested after a period of 4 h. Luciferase activity was determined in triplicate; the activity in the cells treated with 0 mM glucose was set to 100%. Statistical analysis was performed by using Students t-test. *Significant difference p < 0.05. (**b**) INS-1 cells were starved overnight and the next day treated with 0 mM, 5 mM, 11 mM or 25 mM glucose and harvested after a period of 24 h for mRNA isolation with TRIzol® Reagent. *PDX-1* mRNA levels were detected semi-quantitatively by real-time RT-PCR. Fold change of *PDX-1* mRNA expression relative to 0 mM glucose is displayed (mean ± SD, n = 3). (**c**) INS-1 cells were transfected with the PDX-1 promoter construct −6500/+68-STF-luc and the USF1 expression plasmid. After overnight starvation, cells were treated with 0 mM, 5 mM, 11 mM or 25 mM glucose and harvested after a period of 4 hours. Luciferase activity was determined in triplicate; the activity in the 0 mM glucose treated cells was set to 100%. Statistical analysis was performed by using Students t-test. *Significant difference p < 0.05. (**d**) The corresponding Western blot analysis of the FLAG-tagged USF1 is shown beside the graph. Identification of FLAG-tagged USF1 was performed with the mouse monoclonal antibody FLAG M2 (F1804), and α-tubulin served as a loading control. Full-length blots are presented in Supplementary Figure S2.

The transactivation potential of PDX-1 is an important functional determinant for glucose-induced transcription in pancreatic MIN6 β-cells^21^. Although this observation was made with the insulin promoter, we asked whether the transcription of PDX-1 is dependent on the glucose concentration as well. Thus, we transfected INS-1 cells with a reporter construct where luciferase is under the control of the PDX-1 promoter (−6500/+68) and cultivated the starved cells with glucose in concentrations ranging from 0–25 mM for 4 hours. The result of the transactivation experiment is shown in Fig. 3a. We observed an increase in the transactivation efficiency of glucose on the PDX-1 promoter with a 1.5 fold enhancement at 11 mM glucose compared to 0 mM, and somewhat smaller at 5 and 25 mM. This let us to conclude that the PDX-1 transcription is glucose-responsive in INS-1 cells. In a next experiment, we asked whether not only the promoter construct but also endogenous PDX-1 is a target of a glucose-dependent response in INS-1 cells. Thus, we treated cells as described above, but analysed the cells for mRNA of endogenous PDX-1 by quantitative real-time PCR. Relative to the mRNA content, at 0 mM glucose we observed an induction of up to 2.5 fold when increasing the glucose concentration (Fig. 3b). This result perfectly matches the data of the luciferase reporter assay and thus, convinced us that this type of assay represents a reliable tool for further studies.

To investigate the effect of USF1 on the PDX-1 transcription we simultaneously transfected the PDX-1 reporter along with a FLAG-tagged USF1 construct or the empty vector (mock) using different glucose concentrations. Figure 3c shows the luciferase activities after normalization to equal amounts of USF1 as determined by a Western blot analysis with a FLAG epitope specific antibody (Fig. 3d). Compared to a control with the PDX-1 promoter and an empty vector (mock), the transactivation was significantly repressed in the presence of USF1 as already shown in Fig. 1. However, in comparison to the luciferase activity at 0 mM glucose increasing concentrations of glucose attenuated the transrepressing effect of USF1. When normalizing the luciferase activity with 0 mM glucose to 100%, the reporter activity increased by 1.5 fold with 11 mM glucose.

**Figure 4:**
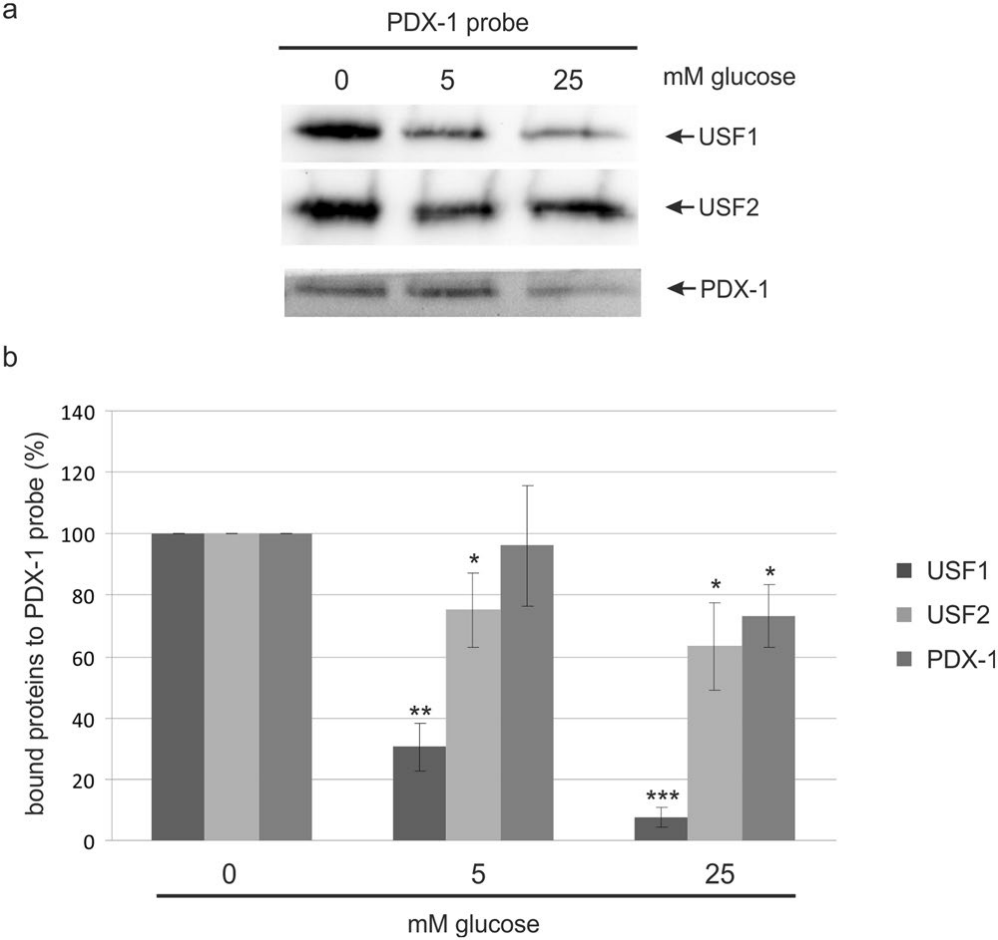
Pull-down assay with the PDX-1 promoter in INS-1 cells treated with glucose. One mg of nuclear extract from INS-1 cells, who had been treated with 0 mM, 5 mM or 25 mM glucose for 4 h, was incubated with 1 μg of a biotinylated PDX-1 DNA probe. The DNA-protein complex was passed through a μMacs™-column and loaded on a 10% SDS polyacrylamide gel for Western blot analysis. (**a**) Identification of USF-binding at the PDX-1 promoter was performed with the USF1 specific antibody sc-8983 and with the USF2 specific antibody sc-862. PDX-1 was visualized with the polyclonal rabbit antiserum against recombinant full-length mouse PDX-1. Full-length blots are presented in Supplementary Figure S2. (**b**) Relative protein amounts of USF1, USF2 and PDX-1 bound to the PDX-1 promoter DNA. The diagram shows the mean ± SD of three independent experiments. Statistical analysis was performed by using Students t-test. *Significant difference p < 0.05, **significant difference p < 0.01, ***significant difference p < 0.001.

Variations in the transactivation or transrepression can be due to an altered DNA binding activity of USFs or PDX-1. Therefore, we analysed the binding of the transcription factors USF1, USF2 and PDX-1 to the proximal PDX-1 promoter. For these DNA pull-down experiments we used a DNA sequence from nt −221 to +22 of the PDX-1 promoter, which contains an E-box element. This E-box element is indispensable for the binding of USF1 and USF2^22^ and detected bound nuclear proteins by Western blot (Fig. 4a); the bar graph summarizes the relative amounts of proteins bound to the PDX-1 probe from three independent experiments (Fig. 4b). Whereas the amount of bound PDX-1 is only weakly, if at all, affected by different glucose concentration, we observed a strong influence on the binding of both USFs to the PDX-1 promoter. With increasing concentration of glucose the binding of USFs is strongly reduced. Thus, we observed a glucose dependent DNA binding of USF at the PDX-1 promoter. This result coincides with the most significant effects of USF in the transactivation assays, with 0 mM glucose we observed the strongest transrepression of USF1 on the PDX-1 promoter (Fig. 3c).

**Figure 5:**
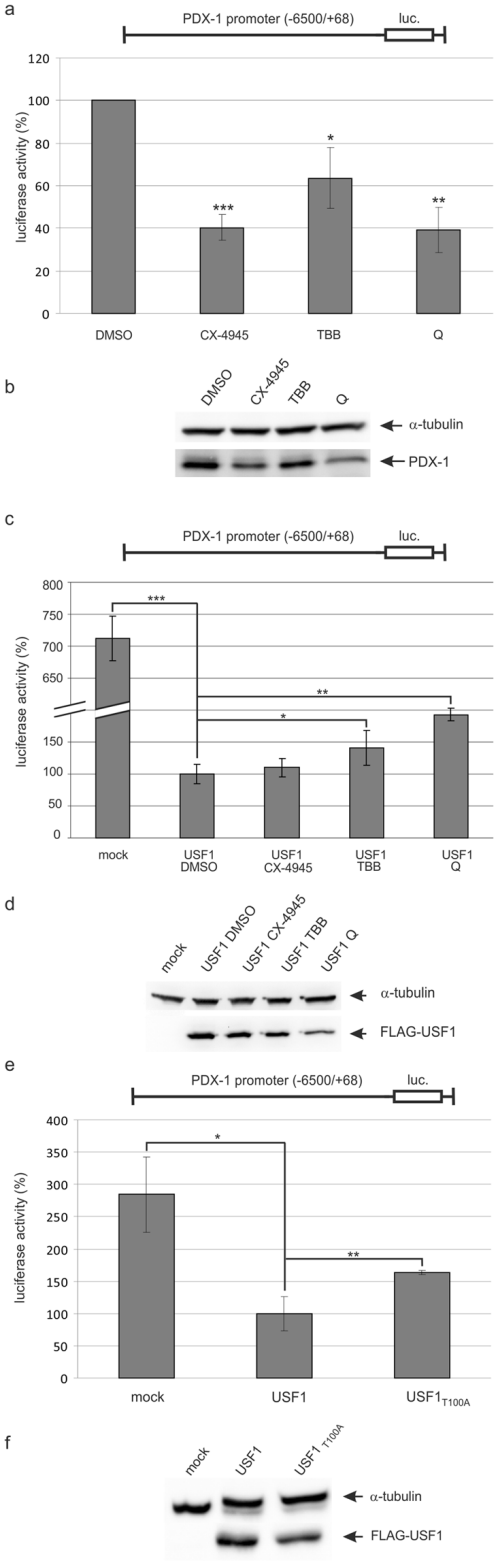
Influence of the CK2 phosphorylation of USF1 on the transactivation of the PDX-1 promoter. (**a**) INS-1 cells were transfected with the PDX-1 promoter construct −6500/+68-STF-luc. After 24 h, cells were treated with 10 µM CX-4945, 50 µM TBB, 50 µM quinalizarin (Q) or with DMSO as a solvent control for 24 h. Luciferase activity was determined in triplicate; the activity in the DMSO treated cells was set to 100%. Statistical analysis was performed by using Students t-test. *p < 0.05, **p < 0.01, ***p < 0.001. (**b**) The corresponding Western blot analysis from one experiment is shown below the graph. PDX-1 was detected with a polyclonal rabbit antiserum, α-tubulin served as a loading control. (**c**) INS-1 cells were transfected with −6500/+68-STF-luc and the FLAG-USF1 or the empty vector as a control. After 24 h, cells were treated with 10 µM CX-4945, 50 µM TBB, 50 µM quinalizarin (Q) or with DMSO as a solvent control for 24 h. Luciferase activity was determined in triplicate; the activity in the USF1 transfected and DMSO treated cells was set to 100%. Statistical analysis was performed by using Students t-test. *p < 0.05, **p < 0.01, ***p < 0.001. (**d**) The corresponding Western blot analysis of the FLAG-tagged USF1 is shown below the graph. FLAG-tagged USF1 was identified with the mouse monoclonal antibody FLAG M2 (F1804), α-tubulin served as a loading control. The relative intensities of FLAG protein bands were normalised first to α-tubulin and then to the luciferase activity. (**e**) INS-1 cells were transfected with −6500/+68-STF-luc and FLAG-USF1, FLAG-USF1_T100A_ or with the empty vector (mock) as a control. After 48 hours, luciferase activity was determined in triplicate; the activity in the mock transfected cells was set to 100%. (**f**) The corresponding Western blot analysis of the FLAG-tagged USF1 is shown below the graph. FLAG-tagged USF1 was identified with the mouse monoclonal antibody FLAG M2 (F1804), α-tubulin served as a loading control. The relative intensities of FLAG protein bands were normalized first to α-tubulin and then to the luciferase activity. Full-length blots are presented in Supplementary Figure S2.

Since both, PDX-1 and USF1, are substrates of protein kinase CK2, in the next step we analysed the influence of the CK2 phosphorylation on both transcription factors. Therefore, we used three different CK2 inhibitors, namely CX-4945, 4,5,6,7-tetrabromobenzotriazole (TBB) and quinalizarin (Q). We used those inhibitors in concentrations, which had previously been tested and found to inhibit the CK2 activity to similar levels^7^. In a first experiment, we checked the influence of these inhibitors upon the reporter activity of the PDX-1 promoter construct. (Fig. 5a). Compared to a solvent treated control (DMSO) the luciferase activity in the treated cells was reduced roughly to 50% by all of three inhibitors. Figure 5b shows the expression of endogenous PDX-1 under these conditions; also the expression of the endogenous PDX-1 was reduced by the treatment with the inhibitors. Thus, inhibition of CK2 activity has a negative impact on the PDX-1 transcription.

To study the role of USF1 under these conditions we transfected INS-1 cells with the FLAG-USF1 construct along with the PDX-1 reporter construct and treated the cells either with the vehicle control DMSO or with CX-4945, TBB or Q for 24 h. Twenty-four hours after treatment we determined the luciferase activities and compared them with the mock-transfected, untreated control. Figure 5c presents the relative luciferase activities after normalization to equal amounts of USF1 (Fig. 5d). As in the previous experiments, we observed a strong repression of the promoter activity after transfection of USF1; the repression turned out to be about sevenfold which is much more distinctive than the inhibitor-dependent reduction. After simultaneous inhibition of CK2 we found a reduced transrepression of USF1. Although all inhibitors showed in general the same result, the best efficiency was obtained with quinalizarin (Q). Thus, simultaneous treatment of USF1-transfected cells with inhibitors seemed to compensate to some extent the strong repressing effect of USF1 upon PDX-1 transcription.

Although, the inhibitors are quite selective for CK2 there is still the possibility that these inhibitors target other kinases or other molecules in the cell. Moreover, some components of the basal transcription machinery are known targets of protein kinase CK2^23,24^ and might also be affected. Therefore, we used a mutant USF1 where we had exchanged the CK2 phosphorylation site at threonine 100 by alanine. We transfected INS-1 cells with the PDX-1 promoter construct alone or together with the FLAG-tagged USF1 wild-type or USF1_T100A_ phospho-mutant construct. We determined the luciferase activities 48 h after transfection and normalized them to equal amounts of FLAG-USF1 protein as shown in the Western blot analysis in Fig. 5f. The relative luciferase activities from three independent experiments are shown in the bar graph in Fig. 5e. As observed before, wild-type USF1 showed a repression of the PDX-1 promoter driven transcription. In contrast, the phospho-deficient T100A mutant led to an attenuation of the transrepression. Thus, using this USF1 mutant we confirmed the results found with the CK2 inhibitors.

**Figure 6:**
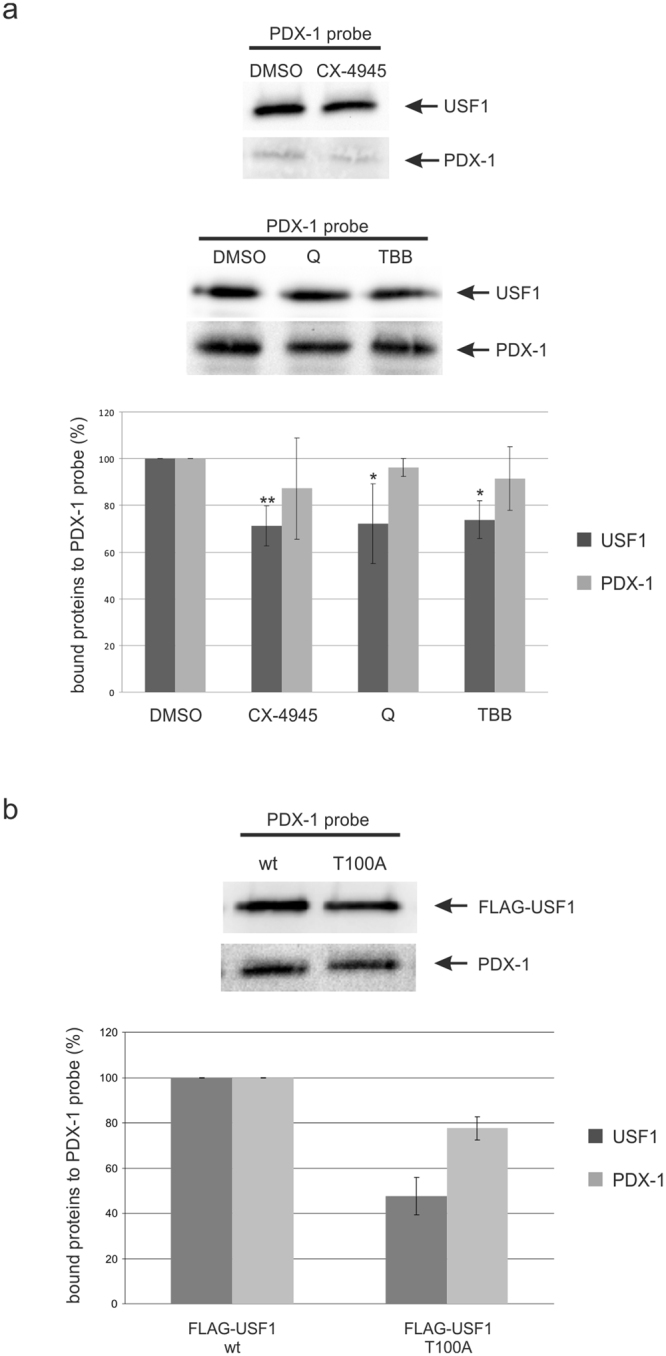
Pull-down assay with the PDX-1 promoter in INS-1 cells treated with CK2 inhibitors or transfected with the phospho-deficient mutant USF1_T100A_. (**a**) One mg of nuclear extract from INS-1 cells treated with 10 µM CX-4945, 50 µM TBB or 50 µM quinalizarin (Q) for 24 h was incubated with 1 μg of the biotinylated PDX-1 DNA probe. The DNA-protein complex was passed through a μMacs™-column and then loaded on a 10% SDS polyacrylamide gel followed by Western blot analysis. Identification of USF1-binding to the PDX-1 promoter was performed with the USF1 specific antibody sc-8983, PDX-1 was visualized with the polyclonal rabbit antiserum against recombinant full-length mouse PDX-1. Relative protein amounts of bound USF1 and PDX-1 were shown in the bar graphs. The diagram shows the mean ± SD of three independent experiments. Statistical analysis was performed by using Students t-test. *Significant difference p < 0.05, **significant difference p < 0.01. (**b**) INS-1 cells were transfected with the wild-type USF1 or with the phospho-deficient mutant USF1_T100A_ for 48 hours. One mg of nuclear extract was incubated with 1 μg of biotinylated PDX-1 DNA probe. The DNA-protein complex was passed through a μMacs™-column and loaded on a 10% SDS polyacrylamide gel for Western blot analysis. Identification of FLAG-USF1-binding at the PDX-1 promoter was performed with the mouse monoclonal antibody FLAG M2 (F1804), PDX-1 was visualized with the polyclonal rabbit antiserum against recombinant full-length mouse PDX-1. Relative protein amounts of bound FLAG-USF1 and FLAG-PDX-1 was shown in the bar graphs. The diagram shows the mean ± SD of three independent experiments. Full-length blots are presented in Supplementary Figure S2.

We now wanted to know whether the reduction in the transrepression of USF1 might be due to altered DNA binding. Therefore, we performed pull-down assays using the PDX-1 probe after treatment of INS-1 with the different CK2 inhibitors. Nuclear extracts from cells grown in the presence of one of the CK2 inhibitors or with DMSO as a control for 24 h were used for DNA pull-down assays. DNA bound PDX-1 and USF1 were detected in a Western blot analysis. Protein amounts from several experiments were quantified by densitometry and presented as bar graph (Fig. 6a). The level of both proteins bound to DNA decreased in the presence of the inhibitors; however, only USF1 showed a significant reduction to about 75% compared to the DMSO control.

To verify the results obtained by the CK2 inhibitors we also used the T100A phospho-mutant of USF1 in comparison to the wild-type USF1. We analysed bound PDX-1 and USF1 bound to DNA by Western blot analysis and observed a reduction of bound USF1_T100A_ to 50% compared to wild-type USF1. The DNA binding of PDX-1 is also diminished, however not as strong as for the USF1 mutant (Fig. 6b). Thus, we have shown with CK2 inhibitors and the CK2 phospho-deficient mutant, that the binding of USF1 to the PDX-1 promoter is reduced in the absence or after reduction of the CK2 phosphorylation of USF1.

**Figure 7:**
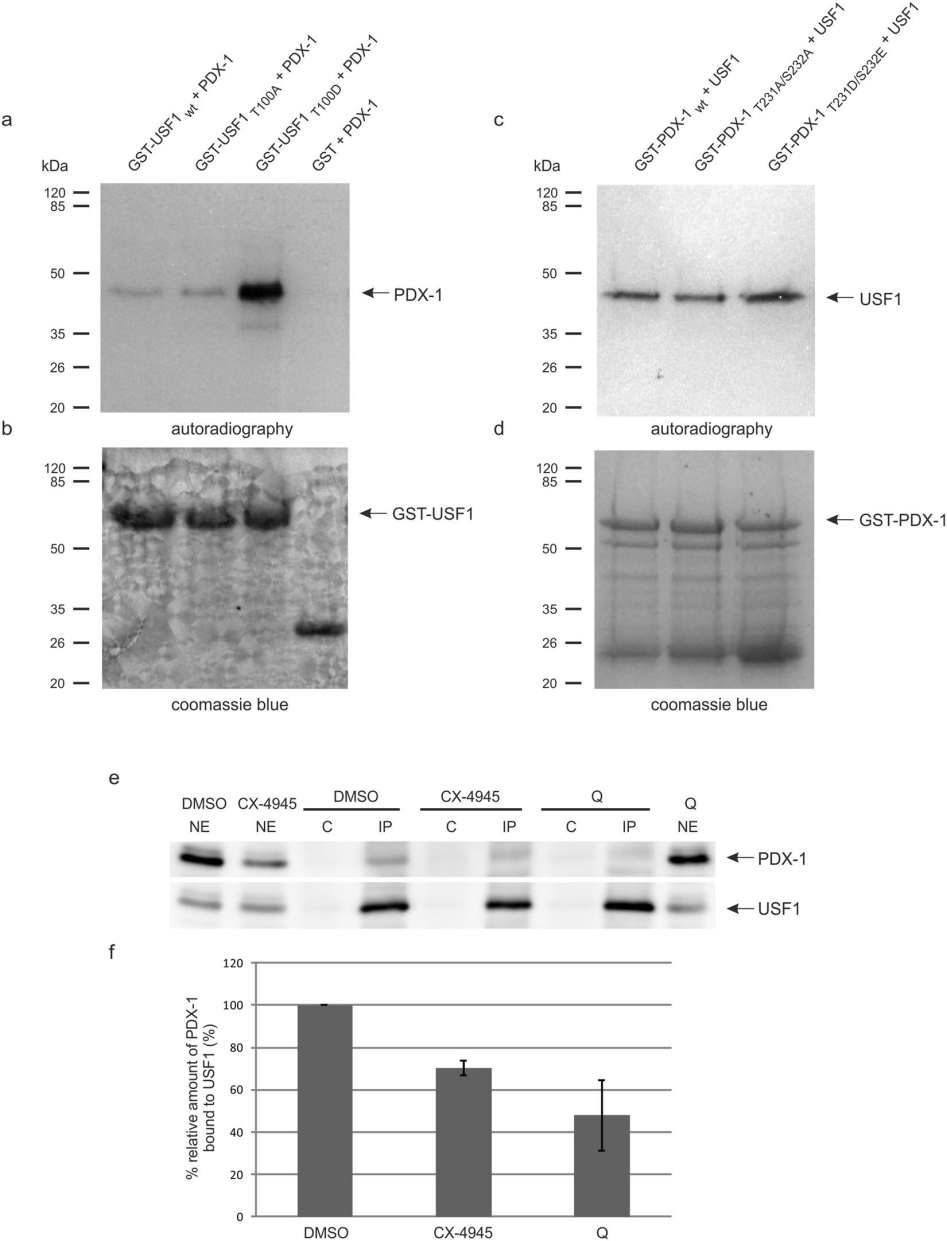
Interaction of PDX-1 and USF1 as a function of CK2 phosphorylation. (**a**–**d**) GST pull-down analysis of the USF1/PDX-1 interaction. (**a**) and (**b**) About 10 µg GST, GST-USF1_wt_, GST-USF1_T100A_ or GST-USF1_T100D_ were incubated with 7.5 µl of *in vitro* translated and [^35^S]methionine labelled PDX-1 protein. The formed complex was coupled to GSH sepharose. Proteins eluted from the affinity resins were analysed on a 12.5% SDS polyacrylamide gel, stained with Coomassie blue (**b**) and afterwards subjected to autoradiography (**a**). (**c**,**d**) About 10 µg GST, GST-PDX-1_wt_, GST-PDX-1_T231A/S232A_ or GST-PDX-1_T231D/S232E_ were incubated with 7.5 µl of *in vitro* translated and [^35^S]methionine labelled USF1 protein. The formed complex was coupled to GSH sepharose. Proteins eluted from the affinity resins were analysed on a 12.5% SDS polyacrylamide gel, stained with Coomassie blue (**d**) and afterwards subjected to autoradiography (**c**). (**e**) INS-1 cells were seeded on a 14.5 cm culture plate and treated with 10 µM CX-4945, 50 µM quinalizarin (Q) or with DMSO as a solvent control for 24 hours. After extraction 2 mg nuclear extract was incubated with the USF1 specific antibody sc-8983 for 2 h. Immunocomplexes were loaded on a 10% SDS polyacrylamide gel and transferred onto a PVDF membrane. Identification of PDX-1 was performed with the polyclonal rabbit antiserum against recombinant full-length mouse PDX-1, detection of USF1 with the USF1 specific antibody sc-8983 served as a control for immunoprecipitation. NE: 50 μg nuclear extract; C: control precipitate; IP: immunoprecipitate. (**f**) Relative protein amounts of bound PDX-1 to USF1 were shown in the bar graphs. The diagram shows the mean ± SD of three independent experiments. The relative intensities of bound PDX-1 protein to USF1 were normalised to the protein content of precipitated USF1. Full-length blots are presented in Supplementary Figure S2.

The auto-regulatory loop model of the concerted interaction of PDX-1 and USF1 and other cofactors at the PDX-1 promoter^16^ suggests an interaction between USF1 and PDX-1, both proteins simultaneously bound to their corresponding binding sites in the PDX-1 promoter. We wondered whether phosphorylation by CK2 might also influence the interaction of both proteins. We first performed GST pull-down assays with wild-type forms or variants where we exchanged the CK2 phosphorylation site to alanine or to the phospho-mimicking aspartic acid. We only observed a weak impact when using USF1 in its wild-type form and different mutants of PDX-1 (Fig. 7c,d). The binding of USF1 to the PDX-1_T231D/S232E_ mutant was about 1.3 fold higher than to the wild-type or alanine double mutant. However, by applying USF1 in wild-type and mutant variants we reproducibly observed a very strong interaction between wild-type PDX-1 and the USF1_T100D_ mutant. Figure 7a shows an autoradiography of bound PDX-1 which was *in vitro* translated in the presence of [^35^S]-methionine; Fig. 7b shows the Coomassie blue staining of GST-USF1 proteins of the same gel. After quantification of five independent experiments the binding turned out to be 3.5 fold better than to the wild-type or the alanine mutant.

To analyse the interaction of both proteins in cells, we treated INS-1 cells with CX-4945 or with quinalizarin, harvested the cells and subjected the nuclear extracts to an immunoprecipitation experiment with an USF1 specific antibody. Under any condition USF1 was precipitated to large amounts (Fig. 7e and f); however, in the presence of CK2 inhibitors the amount of bound PDX-1 decreased to barely detectable levels. Thus, this finding confirms the data from the GST pull-down analysis shown above. Phosphorylation of USF1 by protein kinase CK2 seems to favour its interaction with PDX-1. For CK2 the interaction represents another possibility to intervene in the PDX-1 promoter driven transcription.

**Figure 8:**
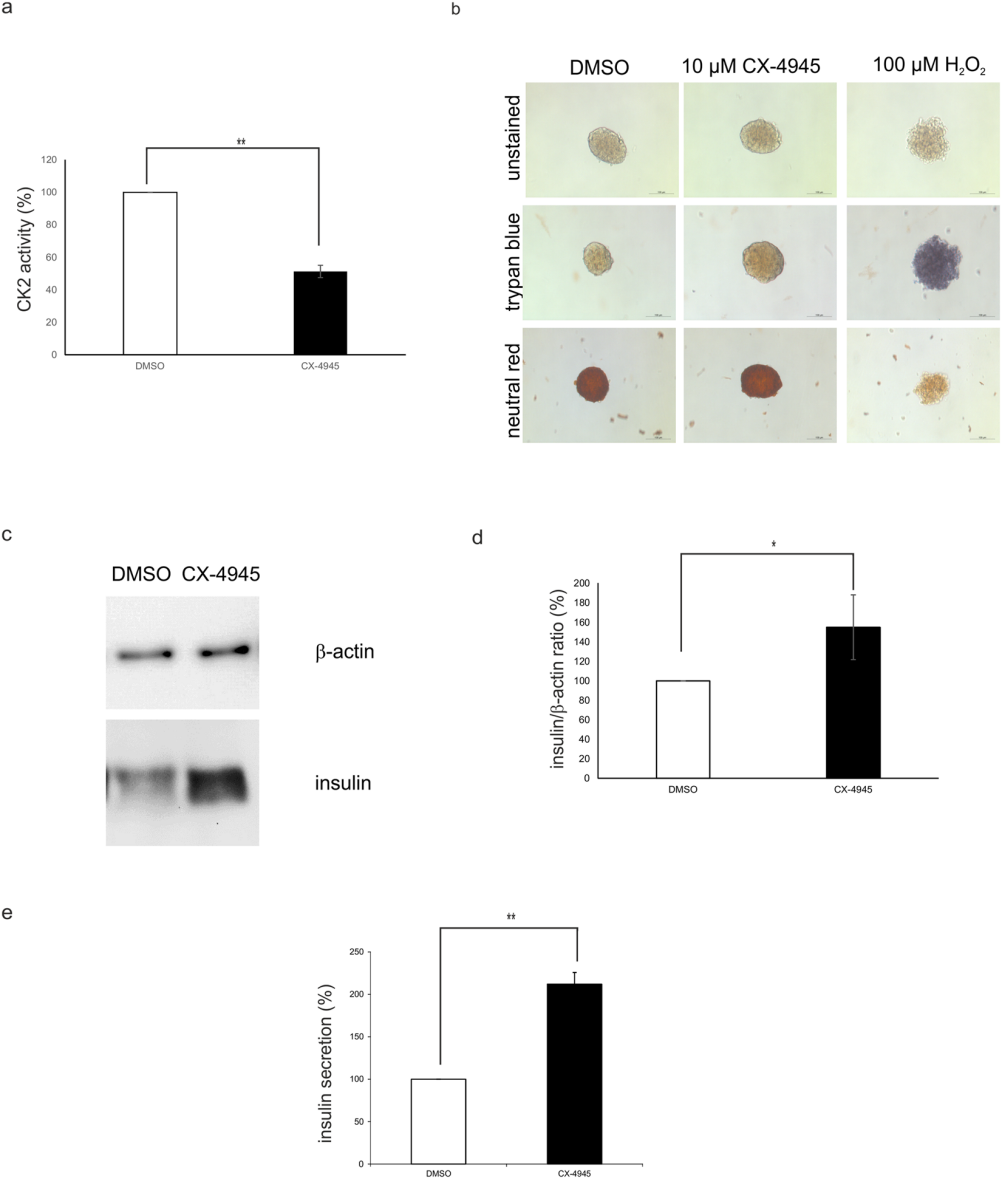
Effect of CK2 inhibition on insulin expression and secretion of isolated pancreatic islets. (**a**) Isolated islets were treated with CX-4945 (10 µM) or DMSO as a solvent control for 24 h. Subsequently, protein extracts were generated and CK2 kinase activity was determined. DMSO treated islets were set 100%. Statistical analysis was performed by using Students t-test, **p < 0.01. (**b**) Light microscopic images of isolated islets which were treated with CX-4945 (10 µM) or DMSO as a solvent control for 24 h. Treatment with 100 µM H_2_O_2_ served as positive control for a treatment with an impact on cell viability. After treatment, islets viability was analysed after trypan blue and neutral red staining with a LEICA DMIL microscope and a LEICA DFC450 C camera. Scale bars: 100 μm. (**c**) Isolated islets were treated with CX-4945 (10 µM) or DMSO as a solvent control for 24 h and the expression of insulin and β-actin was analysed by Western blot. Full-length blots are presented in Supplementary Figure S2. (**d**) Quantitative analysis of (**c**). DMSO-treated islets were used as control and set 100%. Statistical analysis was performed by using Students t-test, *p < 0.05. (**e**) Pancreatic islets were treated with DMSO or 10 µM CX-4945 for 18 hours. After a glucose stimulus secreted insulin was determined in the cell culture supernatant with the rat/mouse insulin ELISA kit. DMSO treated islets were set 100%. Statistical analysis was performed by using Students t-test, **p < 0.01.

To analyse whether CK2 has a significant impact on the functionality of the pancreas, we inhibited CK2 activity in primary islets which were isolated from mouse pancreas. Isolated islets were maintained in cell culture for 24 h in the presence of the solvent control (DMSO) or CX-4945 to inhibit CK2 activity. After treatment, proteins were extracted and CK2 activity was determined with a kinase activity assay. As demonstrated in Fig. 8a, CK2 activity was reduced to about 50% by the application of 10 µM CX-4945. Checking the viability of the islets by staining with trypan blue (dead cells) or with neutral red (living cells) (Fig. 8b), we found that the treatment with the CK2 inhibitor did not influence the viability of cells. Treatment with H_2_O_2_ served as positive control for an agent which reduced the viability of the islets. In a further experiment we checked the effect of CK2 inhibition on the insulin expression and secretion from pancreatic β-cells within the islet. Islets were treated as described before and the insulin expression was determined by Western blot (Fig. 8c and d) as well as the insulin secretion by ELISA (Fig. 8e). We found that after inhibition of CK2, the expression and the secretion of insulin were enhanced about 1.5fold. This indicates that inhibition of CK2 activity in primary islets seems to favour the production and secretion of insulin.

## Discussion

About 30 years ago the *upstream stimulatory factors* USFs were identified as transcriptional regulators of the adenovirus late promoter^25,26^. Two isoforms of USF were isolated as USF1 (43 kDa) and USF2 (44 kDa), which belong to the basic helix-loop-helix leucine zipper (b-HLH-LZ) family of transcription factors^27,28^. Like other b-HLH-LZ transcription factors USFs bind to E-boxes in the promoter region where both prefer CACGTGAC sequence elements. There are opposite results about a growth suppressing or pro-proliferating activity of the USFs^29^. USFs influence cellular proliferation by targeting hTERT, TGFβ2 or IGF2R, cell cycle regulating proteins like cdk4, cyclin B1 or cdk1 or growth suppressor genes like p53, BRCA1 or APC. USFs are stress responsive factors and are active in the tanning and immune response^30^. Besides these effects on cells, USFs are decisively involved in the regulation of glucose and lipid metabolism. Among the USF1-dependent targets of the glucose metabolism are targets of hormonal regulators such as the insulin or glucagon receptor^31,32^, or enzymes of glycolysis like glucokinase^33^ or L-type pyruvate kinase^34^. In addition to these targets the USFs were also found to bind to E-boxes within the promoter of the homeobox gene PDX-1, which plays a key role in the development of the pancreas as well as in the maintenance of pancreatic functions and integrity^22^. In the adult pancreas PDX-1 is responsible for the regulation of the insulin promoter, for GLUT2, as well as for glucokinase expression^35^. PDX-1 regulates its own transcription in an auto-regulatory loop^16^. In addition to the binding of the promoter sequences in the PDX-1 gene the homeobox domain of PDX-1 physically interacts with USFs thus, acting as a cofactor recruiting other cofactors, which are necessary for a successful transcription of the PDX-1 gene^16^. Another example for the co-ordinated action of both transcription factors is the regulation of Alx3 expression in pancreatic β-cells^36^. Alx3 is another homeodomain protein, which has been described to be an important transcription factor for the maintenance of glucose homeostasis^37^. Here, we have shown a functional interaction of USF1 and PDX-1 at the PDX-1 promoter in INS-1 cells. We confirmed the previous observation that PDX-1 acts in an auto-regulatory fashion and transactivates its own promoter. We observed, however, that USF1 had a concentration dependent transrepressing effect on the PDX-1 transcription. This observation seems to be opposite to findings of other groups who showed earlier that an adenovirus mediated overexpression of a dominant negative variant of USF2 (USF2ΔB) decreased the binding of both USF proteins to the E-box motif and this decrease led to a decrease in the PDX-1 gene promoter activity^17^. An explanation for the different result might be that we did not deplete functional USF dimers from INS-1 cells, but we used a controlled over-expression of USF1. Although in most cases USFs are considered as activating transcription factors, there are a few reports that USFs act as transrepressors. This was documented for the human intestinal monocarboxylate transporter 1^38^ and for the catalytic subunit of the glutamate-cysteine ligase (GCLC)^39^. Sharma *et al*. also found that USFs were important components of the PDX-1 transactivating machinery^22^.

As transcription factors are implicated in the maintenance of glucose homeostasis we expected a glucose dependent regulation of the activity and perhaps of the expression of PDX-1 and USFs. It is known for a long time that glucose influences PDX-1 activity, expression and sub-cellular localization, depending on the amount of glucose either positively or negatively^20,21,40–42^. USF1 and USF2 bind to the glucose/carbohydrate response elements (GIRE/ChoRE) that are composed of E-box elements of the CACGTG type which seem to be necessary for the glucose response^43^. Those elements have been identified in the L-type pyruvate kinase promoter and as demonstrated with USF2^−/−^ mice, binding of USF2 to the E-box elements is absolutely required for its glucose-dependent transcription^34^. Another glucose response element has been identified in the rat glucagon receptor gene and it was also demonstrated that USF1/2 are part of the glucose response complex^32^. However, the results concerning the modulation of the binding of USFs to those elements, their transactivation and the expression of USFs depending on the glucose concentration are contradicting and seem to be cell-type and species-specific. Kahn *et al*.^43^ postulated that the binding of USFs to the GRE/ChoRE is not modulated by nutritional conditions in liver. In rat mesangial cells USF1 and USF2 bind to the thrombospondin 1 promoter. Glucose stimulates nuclear USF2 accumulation, which results in an enhanced USF2 level and a higher DNA binding activity to the thrombospondin 1 promoter^11^. The increase in USF2 mRNA and protein expression upon a high glucose stimulus is explained by a glucose-responsive element, which leads to an up-regulation of the CREB-dependent transactivation of the USF2 promoter^44^.

Here, we have shown that glucose increases the transactivation of the PDX-1 promoter by PDX-1 dose-dependently. The glucose-dependent increase in the transcription of PDX-1 was also observed with endogenous PDX-1. With the reporter assay, the strongest effect on transactivation was obtained with 11 mM glucose. With higher concentrations of glucose the effect decreased. This effect is in agreement with a loss of function of PDX-1 under diabetic conditions^45,46^. Furthermore, elevated glucose concentration attenuated the transrepression of the PDX-1 promoter by USF1. Interestingly, an elevated glucose concentration did not influence the DNA binding activity of PDX-1 whereas binding of USF1 and USF2 to the PDX-1 promoter was reduced. These results are consistent with the role of PDX-1 in the regulation of the insulin production after a glucose stimulus. We have shown that this important function of PDX-1 is not counteracted by the transrepressing effect of USF1. Obviously, an elevated glucose concentration has the same effect as inhibition of the CK2 activity. In both cases the transcription of the PDX-1 gene is elevated, which is a prerequisite for an elevated insulin production.

It is well known that DNA binding and transcriptional activity of many transcription factors is regulated by phosphorylation/dephosphorylation. Also PDX-1^4,47^ and USFs (for review see ref.^29^) are phosphoproteins and it is long known that different aspects of their functions can be regulated positively or negatively by phosphorylation. There are several examples that glucose regulates the expression of target genes via kinases or phosphatases. Glucose regulates the APOA5 gene via a dephosphorylation of PP1 and PP2A, which results in a stronger binding of USF1 and USF2 to their promoters^48^. PKG (cGMP dependent protein kinase) inhibits the glucose regulated transcription of the thrombospondin 1 (TSP1) gene via down-regulation of protein levels and DNA binding activity of the TSP1 promoter binding protein USF2; in contrast glucose leads to an enhanced nuclear accumulation of USF2 via PKC, p38 MAPK and ERK pathways^11^.

Over 400 substrates are known for protein kinase CK2, among them different transcription factors whose activity is regulated by CK2 phosphorylation. Phosphorylation by CK2 enhances the transcriptional activity of HIF1α^49^, upstream binding factor UBF^50^, FoxM1c^51^, activating transcription factor 4 ATF4^1^ and C/EBPδ^6^ whereas the transcriptional activity of CHOP is reduced^52^. The function of a transcription factor can switch from a repressing to an activating function by protein kinase CK2, as it was observed for the transcription factor CTCF^53^. CK2 plays a role in the hormonal regulation of carbohydrate metabolism and in modulating activities of enzymes involved in carbohydrate storage and metabolism (for review see ref.^54^). In a recent study we have identified PDX-1 as a CK2 substrate^4^. We have shown that phosphorylation of PDX-1 by CK2 results in a reduction of its transcription factor activity. By using pharmacological inhibition of CK2 activity in pancreatic β-cells, insulin transcription and release was enhanced^4,14^. Here, we have shown that inhibition of CK2 in pancreatic islets from mice led to an elevated production and elevated release of insulin into the culture medium. USF1 was also described as new CK2 substrate^7^. Inhibition of the CK2 kinase activity led to an enhancement of the transcriptional activity towards the fatty acid synthase and the glucokinase promoter^7^. CK2 was not found in the DNA bound USF complex, but an interaction between both proteins was observed in the nucleoplasm^13^. Here, we have demonstrated that inhibition of the CK2 phosphorylation resulted in a decreased transrepression of the PDX-1 promoter. This reduction in the transrepression went along with a reduction in the DNA binding activity of USF1. Pull-down analyses between USF1 and PDX-1 showed a much stronger interaction between PDX-1 and the phospho-mimicking USF1_T100D_ mutant. Thus, it is tempting to speculate that CK2 phosphorylation of USF1 enhances its DNA binding to the PDX-1 promoter as well as its interaction with PDX-1, thus supporting the transrepression activity of USF1. CK2 is likely to act as a negative regulator of the auto-regulatory loop which finally leads to a down-regulation of insulin transcription and release. There are similar observations by Rossi *et al*.^55^ who found that CK2 phosphorylation of the G-protein coupled receptor M3R in pancreatic β-cells impairs its stimulatory effect upon insulin release.

Our results have shown that CK2 acts as a negative regulator of insulin transcription in pancreatic β-cells via the USF-dependent PDX-1 auto-regulatory loop.

## Materials and Methods

### Chemicals and biological reagents

CX-4945 (Selleckchem Munich, Germany)^56^, 4,5,6,7-tetrabromobenzotriazole (TBB, VWR, Darmstadt, Germany)^57^ and quinalizarin (Labotest OHG, Niederschöna, Germany)^58^ were dissolved in dimethyl sulfoxide (DMSO) to 10 mM stock solutions. The TRIzol® Reagent was obtained from Peqlab Biotechnology GmbH (Erlangen, Germany) and the QuantiTect® SYBR® Green RT-PCR kit was purchased from Qiagen GmbH (Hilden, Germany).

### Cell line

INS-1 cells^18^ were cultured in RPMI1640 containing 10% foetal calf serum (FCS), 1% glutamine, 1 mM sodium pyruvate, and 50 µM β-mercaptoethanol. Cells were cultured at 37 °C and 5% CO_2_ in a humidified atmosphere in an incubator.

### Isolation of pancreatic islets

Pancreatic islets of C57B/6 mice (body weight of 25–28 g) were obtained from current animal experiments which are approved by the local governmental animal care committee (Landesamt für Verbraucherschutz, Abteilung C Lebensmittel- und Veterinärwesen, Saarbrücken, Germany) and conducted in accordance with the European legislation on protection of animals (Guide line 2010/63/EU) and the NIH Guidelines for the Care and Use of Laboratory Animals.

The isolation of pancreatic islets was performed as described in Gotoh *et al*.^59^. In brief, animals were anesthetized by intraperitoneal injection of ketamine (75 mg/kg body weight; Ursotamin, Serumwerk Bernburg AG, Bernburg, Germany) and xylazine (15 mg/kg body weight; Rompun, Bayer, Leverkusen, Germany). After laparotomy, the pancreatic duct was injected with collagenase (1 mg/mL, type V, SERVA, Heidelberg, Germany) and the pancreas excised. To isolate the pancreatic islets, the dispersed pancreas was washed in PBS (10% FCS), islets were handpicked and transferred into a Petri dish containing DMEM (10% fetal calf serum, 100 U/ml penicillin and 0.1 mg/ml streptomycin).

### CK2 *in vitro* phosphorylation assay

To determine the kinase activity of CK2, cells from pancreatic islets were lysed with RIPA buffer (50 mM Tris-HCl, pH 8.0, 150 mM NaCl, 0.5% sodium deoxycholate, 1% TritonX-100, 0.1% SDS, complete protease inhibitor mix (Roche diagnostics, Mannheim, Germany) and the extracts were used in a kinase filter assay. In this assay we measured the incorporation rate of [^32^P]- phosphate into the synthetic CK2 specific substrate peptide with the sequence RRRDDDSDDD [21]. Twenty µl kinase buffer (50 mM Tris-HCl, pH 7.5, 100 mM NaCl, 10 mM MgCl_2_, 1 mM DTT) containing 20 µg proteins were mixed with 30 µl CK2 mix (25 mM Tris-HCl, pH 8.5, 150 mM NaCl, 5 mM MgCl_2_, 1 mM dithiothreitol (DTT), 50 µM ATP, 0.19 mM substrate peptide) containing 10 µCi/500 µl [^32^P]γATP. The mixture was spotted onto a P81 ion exchange paper. The paper was washed with 85 mM H_3_PO_4_ for three times. After treatment with ethanol the paper was dried and the Čerenkov-radiation was determined in a scintillation counter.

### Detection of insulin in cell culture supernatant of primary islets

The determination of secreted insulin was essentially done as described by Kelly *et al*.^60^. Five islets from mouse pancreas were maintained in RPMI medium without glucose for 18 hours with 10 µM CX-4945 or an equal volume of DMSO as vehicle control. After washing two times with Krebs Ringer buffer KRB (115 mM NaCl, 4.7 mM KCl, 1.28 mM CaCl_2_,1,2 mM MgSO_4_, 0.1% BSA), islets were incubated 40 min with KRB supplemented with 1.1 mM glucose and either DMSO or 10 µM CX-4945. For another 20 min the glucose concentration was enhanced to 5.6 mM to induce the insulin secretion. After this treatment, insulin was determined in the medium with the rat/mouse insulin ELISA kit from Merck-Millipore (Darmstadt, Germany) according to the recommendation of the provider.

### Plasmids

For eukaryotic expression, USF1 was cloned into the EcoR1 and Xba1 sites of p3xFLAG-Myc-CMV24 thus generating a fusion construct with an N-terminal FLAG-tag and a C-terminal myc-tag. A point mutation of USF1 was created with the p3xFLAG-Myc-CMV24-USF1 and the Q5^®^ Site-Directed Mutagenesis Kit (NEB, Ipswich, USA) to create the p3xFLAG-Myc-CMV24-USF1_T100A_. The mutation was verified by sequencing. For bacterial expression of GST fusion proteins of USF1 wild-type and mutants (T100A, T100D) we used the plasmids described in Lupp *et al*.^7^. For *in vitro* translation of USF1 (pcDNA-USF1) we cloned the cDNA of human USF1 into the EcoR1/Xho1 sites of the pcDNA3.1 vector (Invitrogen). For transient transfection assays p3xFLAG/CMV-7.1-PDX-1 constructs were produced by sub-cloning mouse PDX-1 cDNA into the BamH1 site of p3xFLAG/CMV-7.1 vector^4^. pGEX4T-1-PDX-1 plasmids are described by Meng *et al*.^4^. pRSET-PDX-1 was generated by cloning the cDNA of mouse PDX-1 in frame into the BamH1/HindIII sites of the pRSETA plasmid (Invitrogen).

The PDX-1 promoter construct from −6500 to +68^22^ was a kind gift from Dr. Marc R. Montminy (La Jolla, USA). The dominant-negative USF mutant CMV566 A-USF was a gift from Charles Vinson (Addgene plasmid # 33360).

### Antibodies

The anti-USF1 antibody is a rabbit polyclonal IgG directed against the C-terminus of USF1 (c-20, sc-229) or a rabbit polyclonal IgG directed against the amino acids 75–160 of USF1 (H-86, sc-8983), whereas the anti-USF2 antibody is a rabbit polyclonal IgG directed against the C-terminus of USF2 (c-20, sc-862). HA-tagged dominant negative USF mutant was detected with the HA specific mouse monoclonal antibody 12CA5 from Roche Diagnostics (Mannheim, Germany). PDX-1 was identified with a polyclonal antiserum generated by immunizing rabbits with recombinant mouse PDX-1^20,61^. The mouse monoclonal antibody against CK2β (E-9, sc-46666) was purchased from Santa Cruz (Biotechnology Inc., Heidelberg, Germany). Detection of CK2α was performed by using the mouse monoclonal antibody 1 A5^62^. The mouse monoclonal antibody FLAG M2 (F1804), the β-actin and the α-tubulin (clone DM1A) antibody were from Sigma-Aldrich (Munich, Germany). Rabbit polyclonal serum #36 against nucleolin was prepared in our laboratory^63^. Insulin was detected by the monoclonal rabbit antibody AB181547 from abcam (Cambridge, UK).

### Transfection, treatment and luciferase assay

Transfection of cells was performed by using the Viafect^TM^ Transfection Reagent (Promega, Mannheim, Germany) according to the manufacturer’s instructions.

For the luciferase reporter assay, INS-1 cells were seeded into a 6-well plate in a total volume of 2 ml/well of cell culture medium and cultured overnight. Cells were then transfected with 2.5 µg plasmid DNA by using Viafect^TM^ Transfection Reagent. 24 h after transfection cells were starved overnight for glucose experiments and the next day treated with different glucose concentrations (0 mM, 5 mM, 11 mM or 25 mM) over a period of 4 h. For glucose and/or parallel treatment with the CK2 inhibitors we used CX-4945 in a concentration of 10 µM, whereas TBB and quinalizarin were used in a concentration of 50 μM over a period of 4 h or 24 h.

4 h or 24 h after treatment or 48 h after transfection cells were collected by lysing in 1x lysis buffer (LB, Promega, Mannheim, Germany) and measured with the Luciferase Reporter Assay System (Promega) following the manufacturer’s recommendations. To normalize the experimental variations such as differences in transfection efficiencies we calculated the relative intensities of FLAG tagged protein amounts first in relation to α-tubulin and then in relation to the luciferase activity.

For Western blot analysis, cell lysates were centrifuged at 13.000xg to remove cell debris. The protein content was determined with BioRad reagent dye (BioRad, Munich, Germany). Protein extracts were immediately used for Western blot analysis or stored at −20 °C. Image acquisition of Western blots was performed with the ChemiDoc XRS+ imaging system from Bio‐Rad (Bio‐Rad Laboratories GmbH, München, Germany); data were analyzed using the Quantity One 1‐D analysis software, version 4.6.

### Co-immunoprecipitation of USF1 and PDX-1

For co-immunoprecipitation of USF1 and PDX-1, INS-1 cells were seeded on a 14.5 cm cell culture plate in a total volume of 12 ml/plate of culture medium. After a period of 24 h cells were starved overnight and the next day treated with glucose at different concentrations (0 mM, 5 mM or 25 mM) over a period of 4 h. After 4 h nuclear proteins were extracted. For CK2 inhibition, cells were treated with 10 µM CX-4945 or 50 µM quinalizarin (Q) over a period of 24 h and then nuclear proteins were extracted. For extraction, cells were washed two times with PBS (137 mM NaCl, 2.7 mM KCl, 8 mM Na_2_HPO_4_, 1.5 mM KH_2_PO_4_, pH 7.4), resuspended in buffer A (10 mM HEPES, pH 7.9, 1.5 mM MgCl_2_, 10 mM KCl, 0.5 mM DTT, Complete™) and incubated on ice for 20 min. After centrifugation, the supernatant containing cytoplasmic proteins was removed and the pellet was resolved in buffer C (20 mM HEPES, pH 7.9, 1.5 mM MgCl_2_, 0.2 mM EDTA, 0.5 mM DTT, Complete™). Nuclear proteins were isolated by pressing the suspension through a 21 G and 23 G cannula and centrifugation (13.000 × g, 30 min, 4 °C). Two mg of nuclear proteins were pre-cleared twice with protein G sepharose beads (GE Healthcare, Freiburg, Germany) (control precipitate). The supernatant was incubated with rabbit polyclonal antibody USF1 (sc-8983, 4 µg) for 2 h at 4 °C. Beads were washed four times with PBS (137 mM NaCl, 2.7 mM KCl, 8 mM Na_2_HPO_4_, 1.5 mM KH_2_PO_4_, pH 7.4), and bound proteins were analysed by Western blot with specific antibodies. Image acquisition of Western blots was performed with the ChemiDoc XRS+ imaging system from Bio‐Rad (Bio‐Rad Laboratories GmbH, München, Germany); data were analyzed using the Quantity One 1‐D analysis software, version 4.6.

### Streptavidin-agarose pull-down assay with the PDX-1 promoter

For *in vitro* analysis of binding of the USF proteins and PDX-1 to sequence-specific regulatory elements on the PDX-1 promoter regions, we used the μMacs^TM^ Streptavidin Kit (Miltenyi Biotec, Gladbach, Germany).

Several biotinylated primers were obtained from Integrated DNA Technologies (Life Technologies, Darmstadt). We used the forward primer 5^′^-CAGGACAGGAGAGATCAGCC-3^′^ and the biotinylated reverse primer 5^′^-biotin/GCAGCTCACGGACTCTCA-3^′^ for PCR amplification to prepare a biotinylated PDX-1 promoter probe from −221 to +22.

For *in vitro* pull-down assays with the PDX-1 promoter, INS-1 cells were seeded on a 14.5 cm cell culture plate in a total volume of 12 ml/plate of culture medium. After a period of 24 hours, cells were starved overnight and the next day treated with different glucose concentrations (0 mM, 5 mM or 25 mM) over a period of 4 h. For treatment with the CK2 inhibitors we used CX-4945 in a concentration of 10 µM, whereas TBB and quinalizarin were used in a concentration of 50 μM over a period of 24 h.

Nuclear extracts were prepared as described above and 1 mg was incubated with salmon sperm DNA (100 µg/ml) for 20 minutes at 4 °C. One µg of biotinylated PDX-1 DNA probe was added and the extract was again incubated for 30 minutes at 4 °C. 50 µl of streptavidin-agarose beads were added for 12 minutes at room temperature and the DNA-protein complex was passed through an equilibrated μMacs^TM^-column. The column was washed 15 times with buffer C (150 mM NaCl) and the proteins were eluted by the addition of 50 µl pre-heated SDS sample buffer and after SDS polyacrylamide gel electrophoresis analysed by Western blot with specific antibodies. Image acquisition of Western blots was performed with the ChemiDoc XRS+ imaging system from Bio‐Rad (Bio‐Rad Laboratories GmbH, München, Germany); data were analyzed using the Quantity One 1‐D analysis software, version 4.6.

### GST pull-down assay

The pull-down assay was essentially done as described by Sun *et al*.^64^. Purified GST- or GST-tagged proteins (10 μg) were immobilized on GSH-sepharose and equilibrated with PBS-T binding buffer (PBS, pH 7.4, 1% Tween 20). Immobilized proteins were incubated for 2 h at 4 °C with 7.5 µl of PDX-1 product from an *in vitro* translation reaction. The translation from a pRSET-PDX-1 plasmid was done as recommended by the manufacturer (Promega). After washing with cold PBS-T, bound proteins were eluted with SDS sample buffer (65 mM Tris-HCl, pH 6.8, 2% SDS, 5% β-mercaptoethanol, 10% glycerol, 0.01% bromophenol blue) and analysed by SDS polyacrylamide gel electrophoresis, followed by protein staining with Coomassie blue and autoradiography.

### mRNA Extraction and real-time RT-PCR

Total RNA and protein were isolated using TRIzol® Reagent (peqlab biotechnology, Erlangen, Germany) following the manufacturer’s protocol. One-step real-time RT-PCR was performed as described previously^65^ employing the LightCycler 2.0 instrument (Roche, Mannheim, Germany) using the QuantiTect® SYBR® Green RT-PCR kit (Qiagen, Hilden, Germany). The following primers were used for real-time RT-PCR: PDX-1_for: 5′-AGCGAGATGCTGGCAGA-3′ and PDX-1_rev: 5′-TCAGTTGGGAGCCTGATTCT-3′. 18 S rRNA was used as an endogenous control employing the primers 18S_for: 5′-GTAACCCGTTGAACCCCATT-3′ and 18S_rev: 5′-CCATCCAATCGGTAGTAGCGA-3′.

### Statistical analysis

Microsoft Excel 2007 and the software “Quantity one 1-D Analysis” from Bio-Rad Laboratories Inc. were used to analyse the data. Results from the luciferase assay were expressed as mean ± standard deviation (SD) of at least three or four independent experiments. Statistical analysis of the data was performed using the student’s t-test (two-tail, paired), statistical significant differences were shown as follows: ***p < 0.001, **p < 0.01 or *p < 0.05.

## Electronic supplementary material

Supplementary figure S1
