# Supplementary material for: Functional interplay between the transcription factors USF1 and PDX-1 and protein kinase CK2 in pancreatic β-cells
Source: Sci Rep. 2017 Nov 27;7:16367. doi: 10.1038/s41598-017-16590-0 (PMC5703852; doi:10.1038/s41598-017-16590-0)

Functional interplay between the transcription factors USF1 and PDX-1 and protein kinase CK2 in pancreatic β-cells

Sarah Spohrer[1], Rebecca Groß[1,#], Lisa Nalbach[2,#], Lisa Schwind[1], Heike Stumpf[1], Michael D. Menger[2], Emmanuel Ampofo[2], Mathias Montenarh[1] and Claudia Götz[1]*

[1] Medical Biochemistry and Molecular Biology, Saarland University, Homburg, Germany

[2] Institute for Clinical & Experimental Surgery, Saarland University, Homburg, Germany

Supplementary figure S1: Cell fractionation of glucose treated INS-1 cells

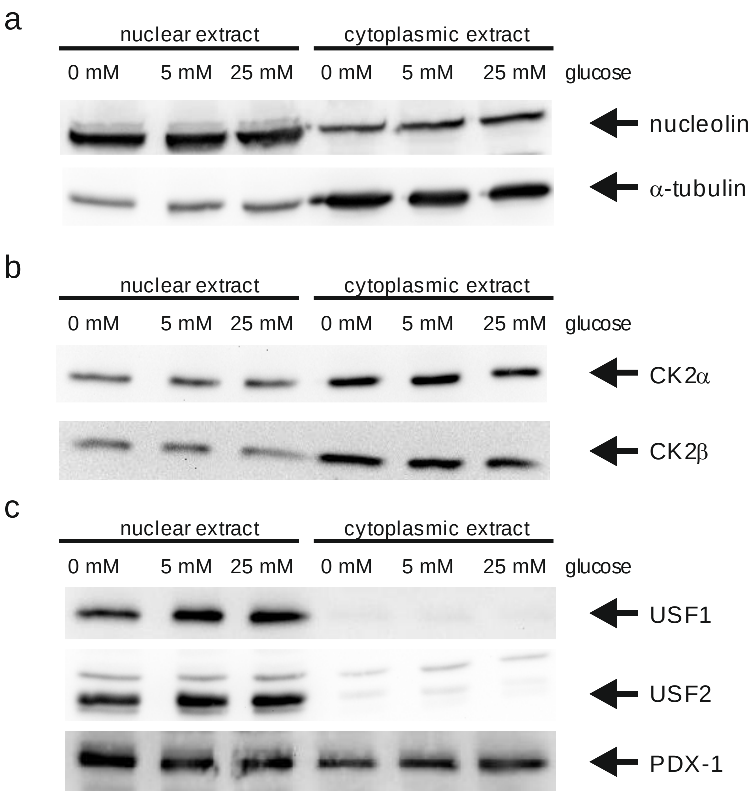

INS-1 cells were seeded on a 14.5 cm culture plate and starved overnight. The next day, cells were treated with 0 mM, 5 mM or 25 mM glucose and after a period of 4 h cytoplasmic and nuclear proteins were extracted as described in material and methods. Fifty µg of each fraction was loaded on a 12.5% SDS polyacrylamide gel and transferred on a PVDF membrane. (a) The nuclear protein nucleolin was detected with rabbit polyclonal serum #36, α-tubulin was used as a control for cytoplasmic proteins, it was visualized with the mouse monoclonal antibody clone DM1A. (b) CK2α was detected with the mouse monoclonal antibody 1A5 and CK2β was visualized with the mouse monoclonal antibody clone E-9. (c) Identification of the USF-proteins was performed with the rabbit polyclonal antibody USF1 (sc-8983) and the rabbit polyclonal antibody USF2 (sc-862). The same extracts were loaded on a separate gel for the detection of PDX-1; PDX-1 was detected with the polyclonal rabbit antiserum against recombinant full length mouse PDX-1.

Unprocessed original scans of western blots shown in figures 1 – 8 and supplementary figure S1

Fig. 1 Reporter assays after transfection of INS-1 cells with USF1 and PDX-1 at different concentrations**.**

Figure 1b upper part
first antibody anti USF1 (sc8983)

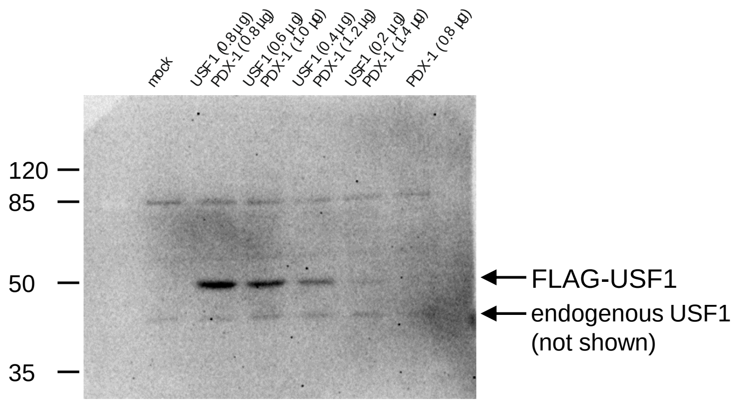

Figure 1b upper part
Same blot; second antibody anti-tubulin

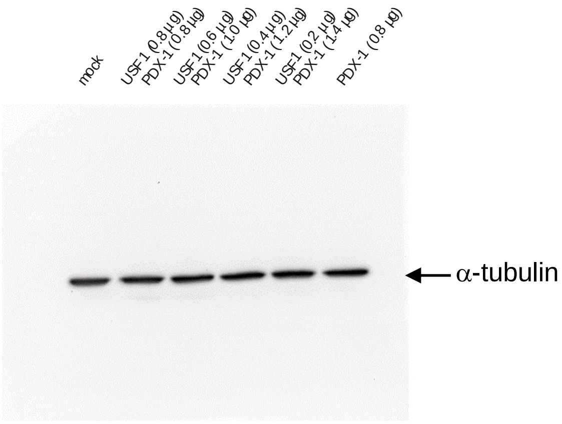

Same extracts loaded on another gel:

Figure 1b lower part
first antibody anti PDX-1

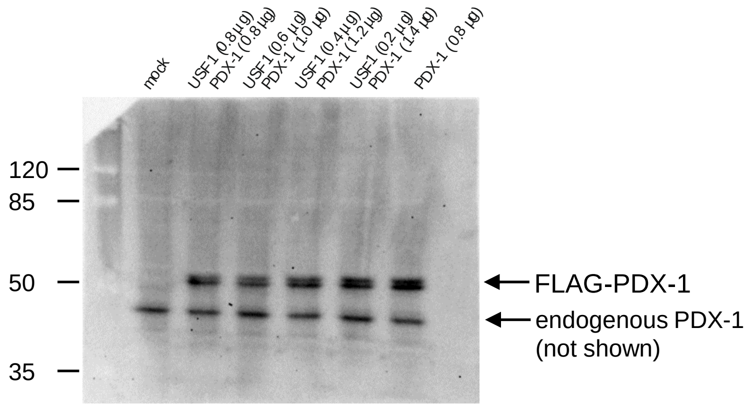

Figure 1b lower part
Same blot; second antibody anti-tubulin AK

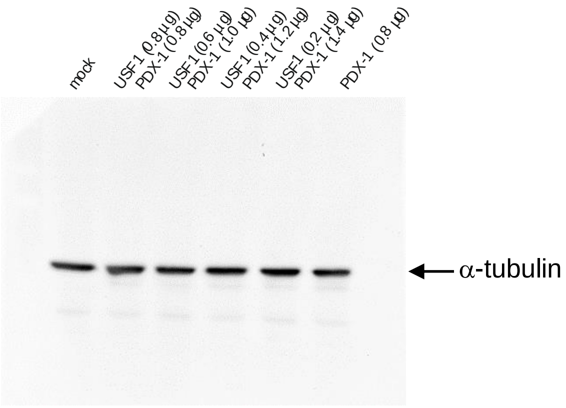

Fig. 1 Reporter assays after transfection of INS-1 cells with USF1 and PDX-1 at different concentrations**.**

Figure 1d upper part
first antibody anti USF1 (sc8983)

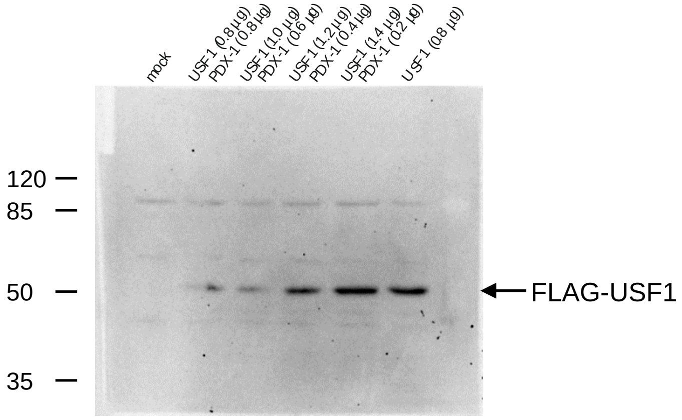

Figure 1d upper part
Same blot; second antibody anti-tubulin

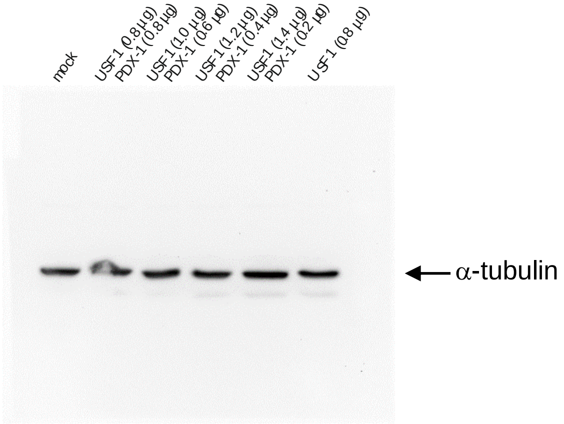

Same extracts loaded on another gel:

Figure 1d lower part
first antibody anti PDX-1

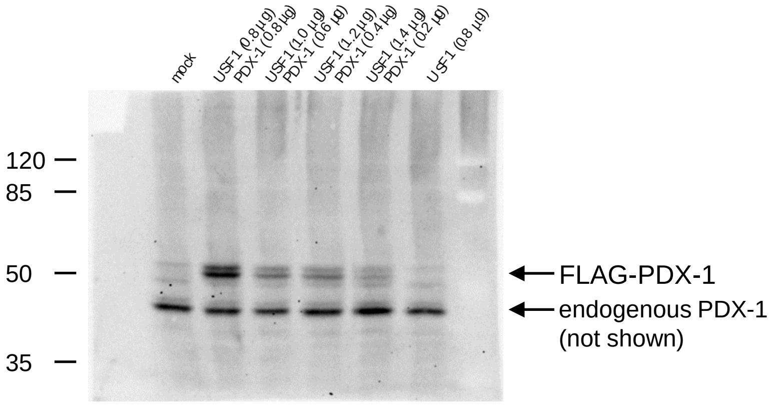

Figure 1d lower part
Same blot; second antibody anti-tubulin

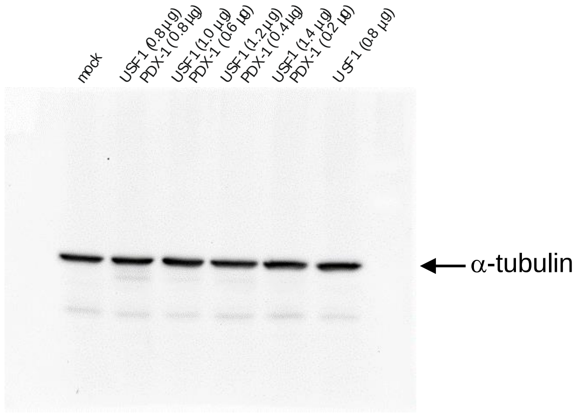

Fig. 1 Reporter assays after transfection of INS-1 cells with USF1 and PDX-1 at different concentrations**.**

Figure 1f upper part
first antibody anti USF1 (sc8983)

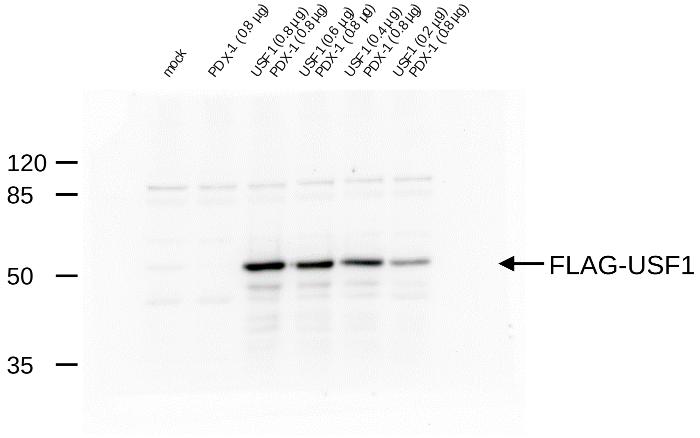

Figure 1f upper part
Same blot; second antibody anti-tubulin

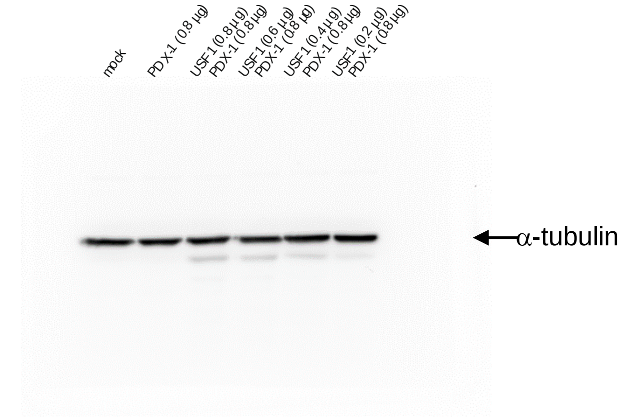

Same extracts loaded on another gel:

Figure 1f lower part
first antibody anti PDX-1

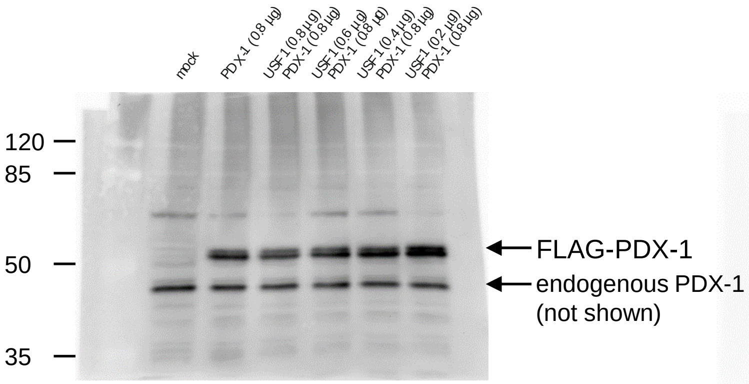

Figure 1f lower part
Same blot; second antibody anti-tubulin

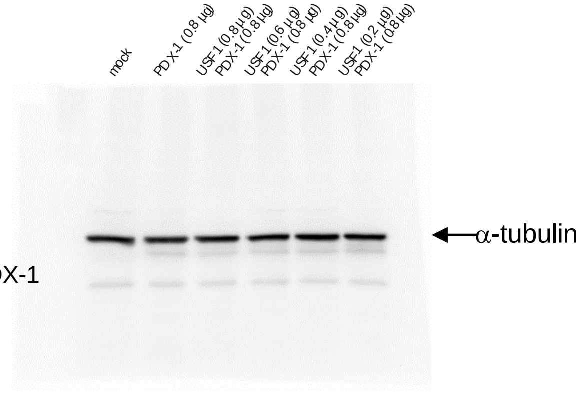

Fig. 1 Reporter assays after transfection of INS-1 cells with USF1 and PDX-1 at different concentrations**.**

Figure 1 h
antibody anti HA 12CA5

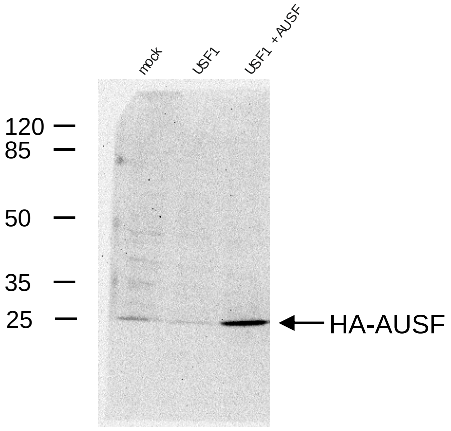

Figure 1h
Same blot, second antibody anti FLAG

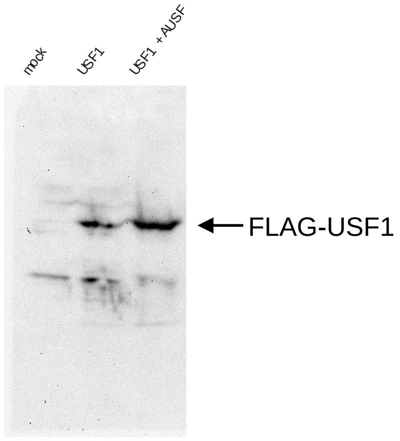

Same extracts loaded on another gel:

Figure 1h
same samples, lower amount , anti tubulin

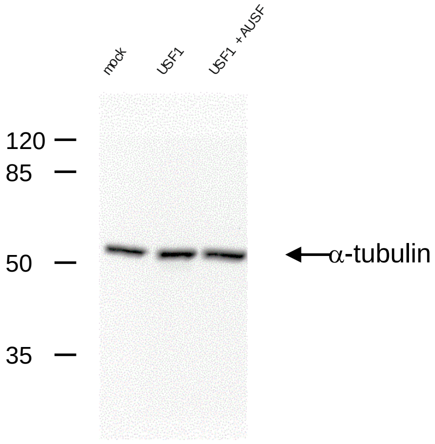

Fig. 2 Co-immunoprecipitation of PDX-1 and USF1

Figure 2a
First antibody anti PDX-1 → crossreactivity with IgG heavy chains from antibody used for immunoprecipitation → blot cut (upper part not shown)

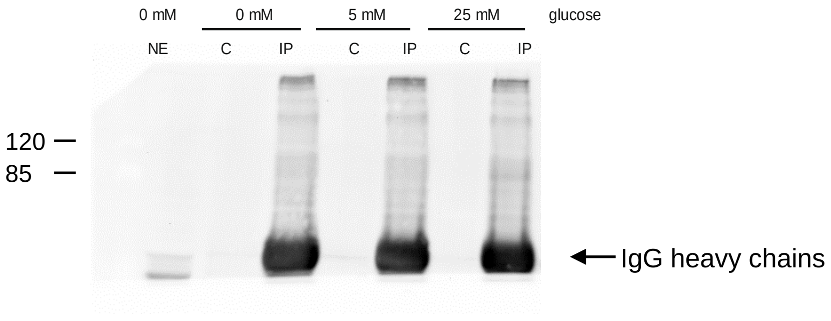

Figure 2a
First antibody anti PDX-1 → signal after covering upper part

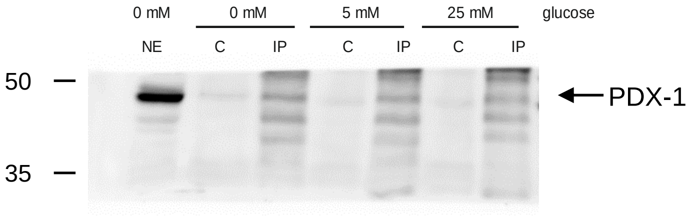

Figure 2a
Second antibody anti USF1 (sc8983)

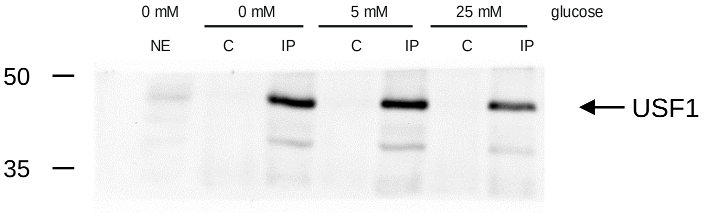

Fig. 3 Influence of glucose on the transcription of the PDX-1 promoter of INS-1 cells

Figure 3d
first antibody anti FLAG

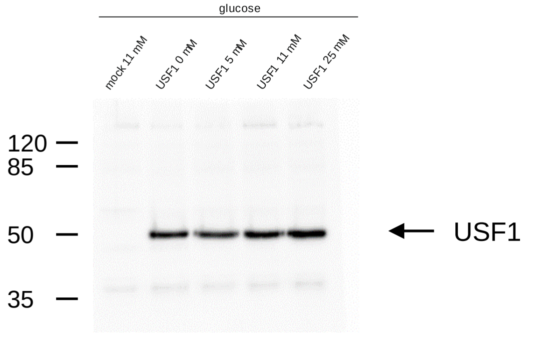

Figure 3d
second antibody anti tubulin

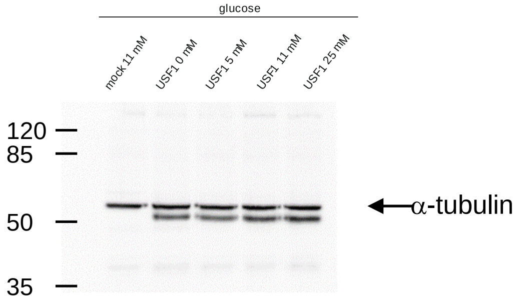

Fig. 4 Pull-down assay with the PDX-1 promoter in INS-1 cells treated with glucose

Figure 4d
First antibody anti USF1 (sc8983)

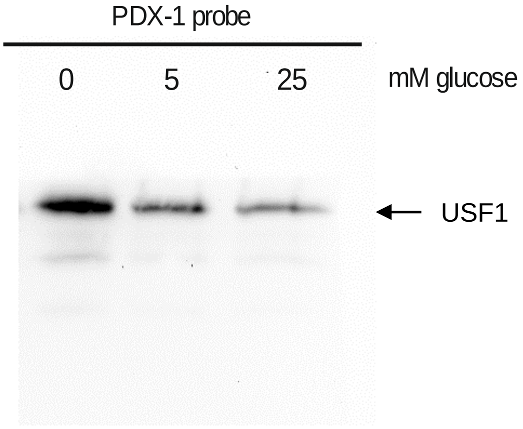

Figure 4d
Same extracts, loaded on another gel, anti PDX-1

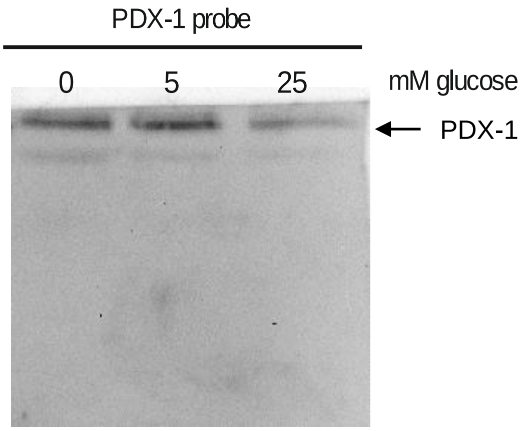

Figure 4d
Samples from a different experiment, anti USF2
(sc-862)

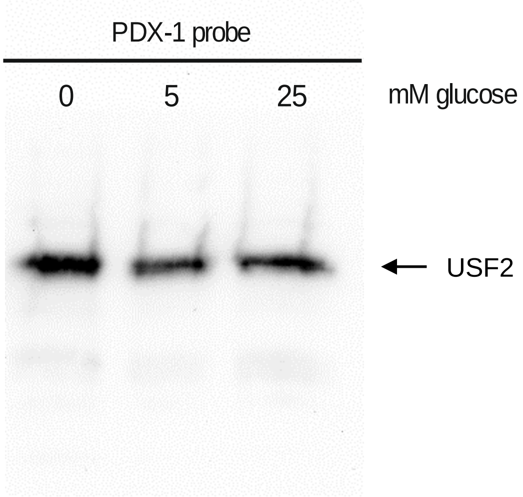

Fig. 5 Influence of the CK2 phosphorylation of USF1 on the transactivation of the PDX-1 promoter

Figure 5b
first antibody anti PDX-1

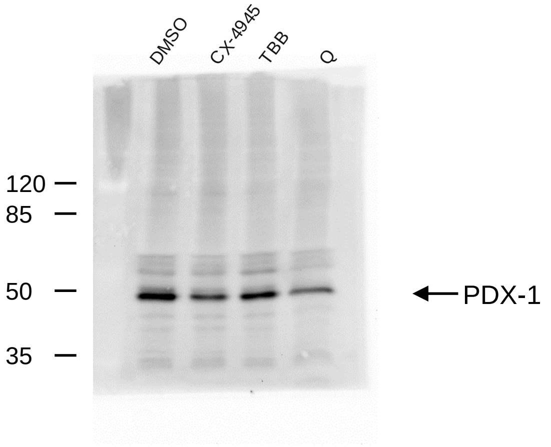

Figure 5b
second antibody anti tubulin

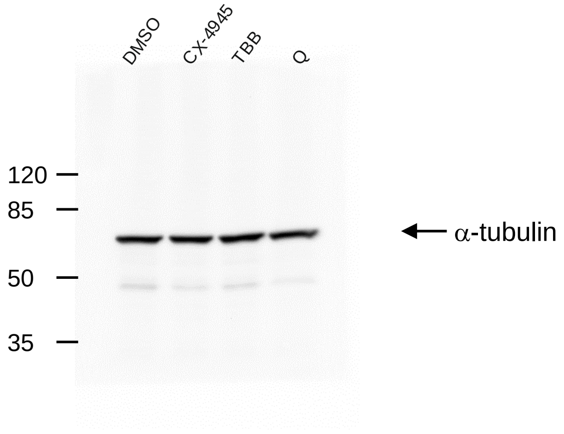

Fig. 5 Influence of the CK2 phosphorylation of USF1 on the transactivation of the PDX-1 promoter

Figure 5d
first antibody anti FLAG

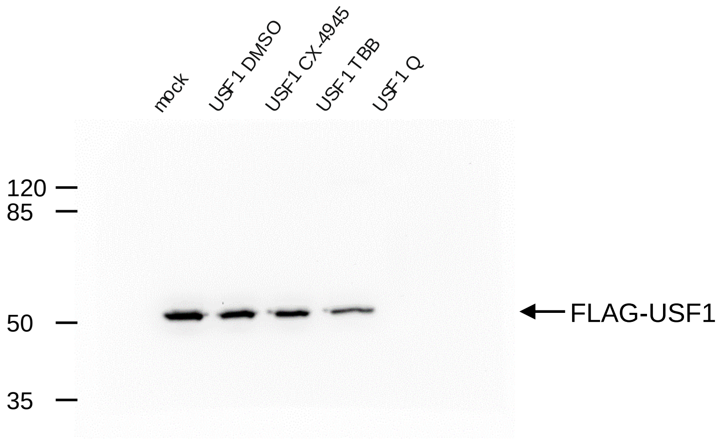

Figure 5d
second antibody anti tubulin

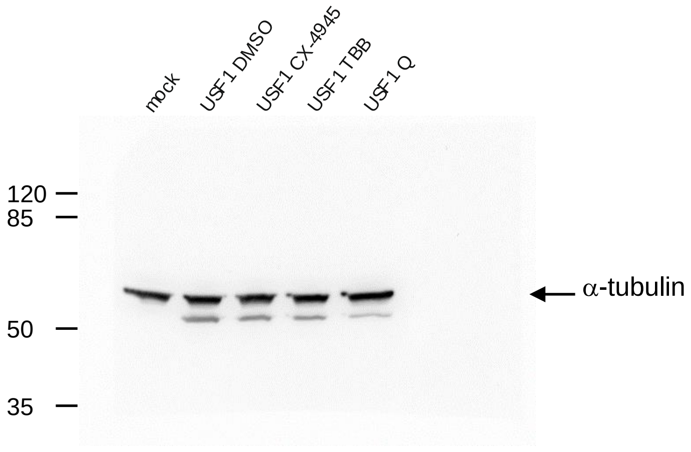

Fig. 5 Influence of the CK2 phosphorylation of USF1 on the transactivation of the PDX-1 promoter

Figure 5f
first antibody anti FLAG

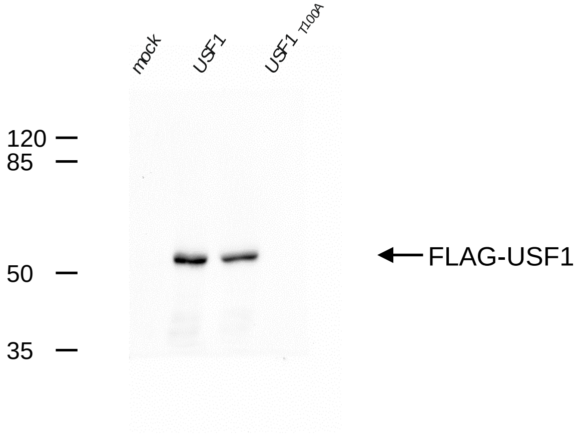

Figure 5f
second antibody anti tubulin

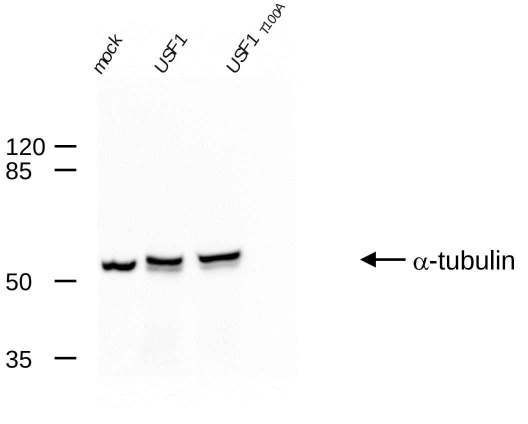

Fig. 6 Pull-down assay with the PDX-1 promoter in INS-1 cells treated with CK2 inhibitors or transfected with the phospho-deficient mutant $USF1_{T100A}$.

Figure 6a upper part
anti USF1
In the manuscript the middle two lanes are shown

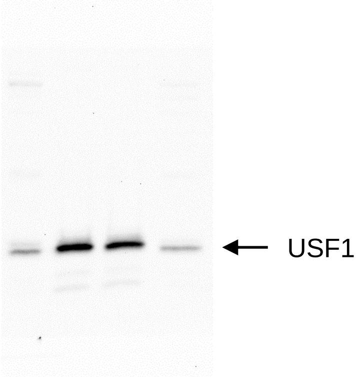

Figure 6a upper part
anti PDX1
In the manuscript the middle two lanes are shown

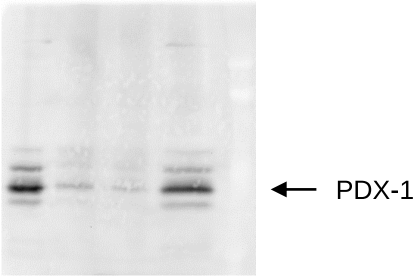

Figure 6a lower part
anti USF1

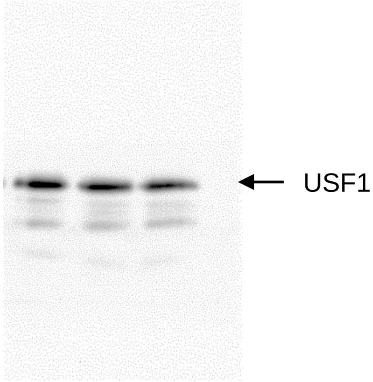

Figure 6a lower part
anti PDX1

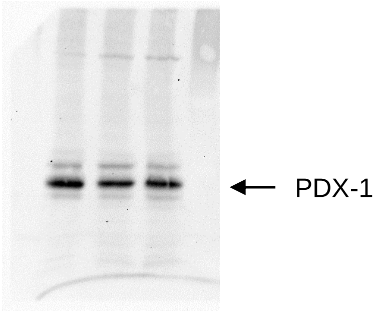

Figure 6 b
anti USF1
In the manuscript the middle two lanes are shown

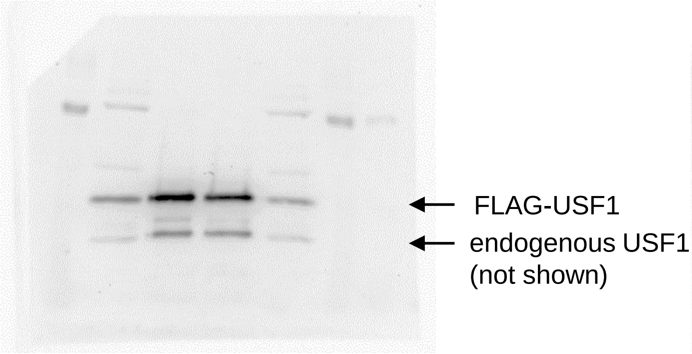

Figure 6 b
anti PDX1
In the manuscript the middle two lanes are shown

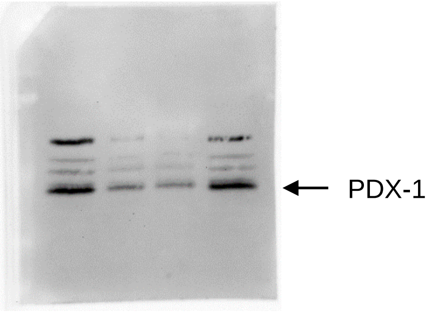

Fig. 7 Interaction of PDX-1 and USF1 as a function of CK2 phosphorylation.

Figure 7e first antibody anti PDX-1
Upper half of the blot was covered because of cross reactivity with IgG heavy chains (see second antibody)

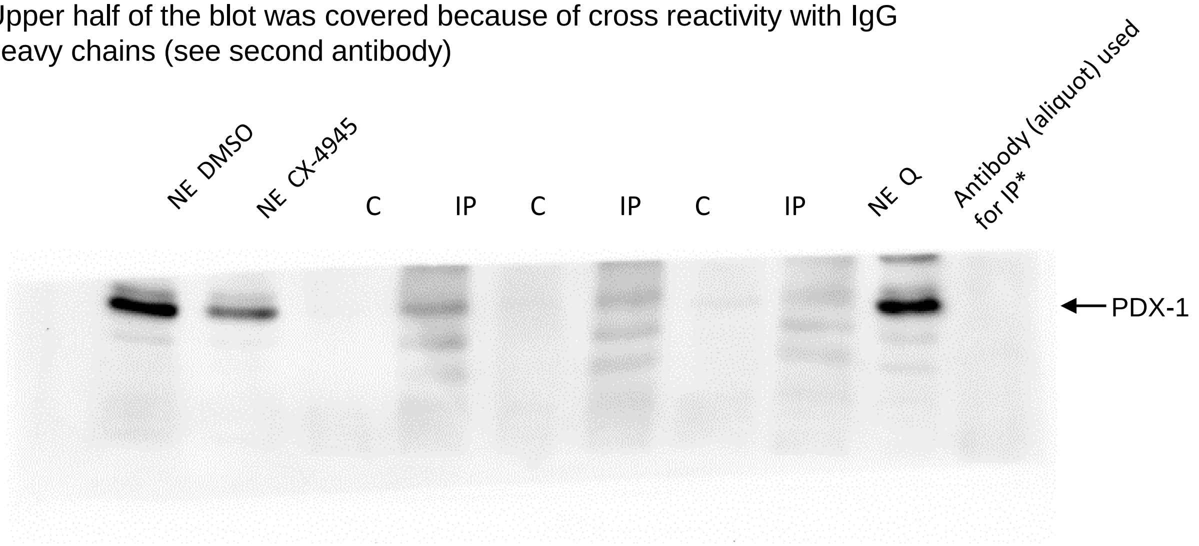

Figure 7e
second antibody anti USF1

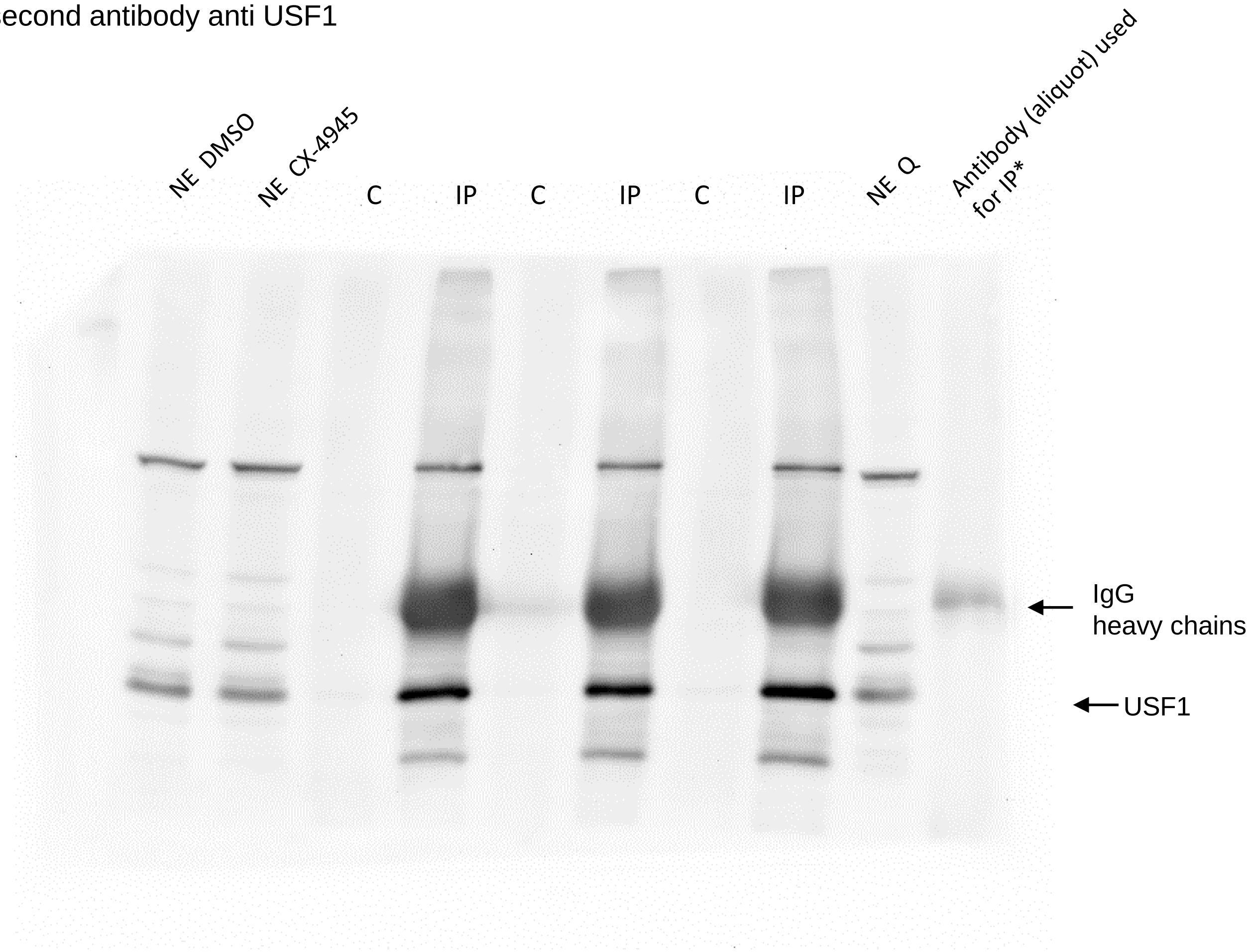

* Lane not shown in the manuscript

Fig. 8 Effect of CK2 inhibition on insulin expression and secretion of isolated pancreatic islets.

Figure 8c
first antibody anti insulin

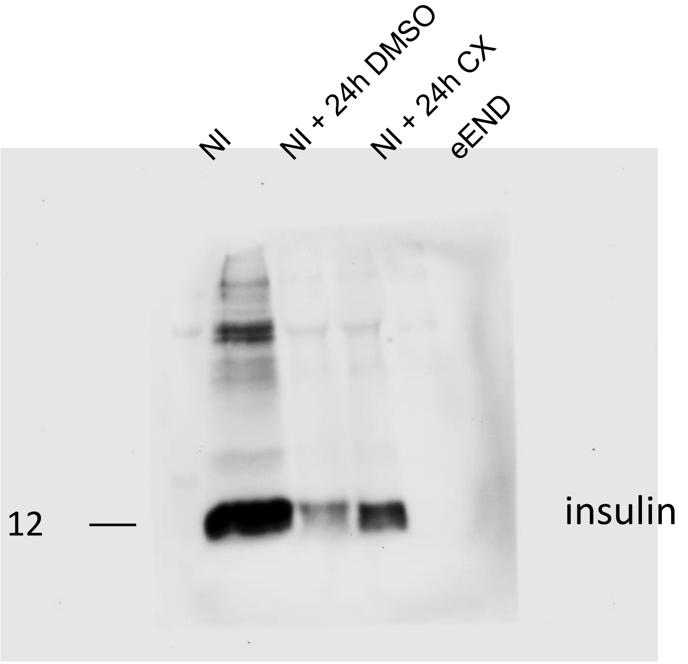

Figure 8c
Same blot, second antibody anti actin

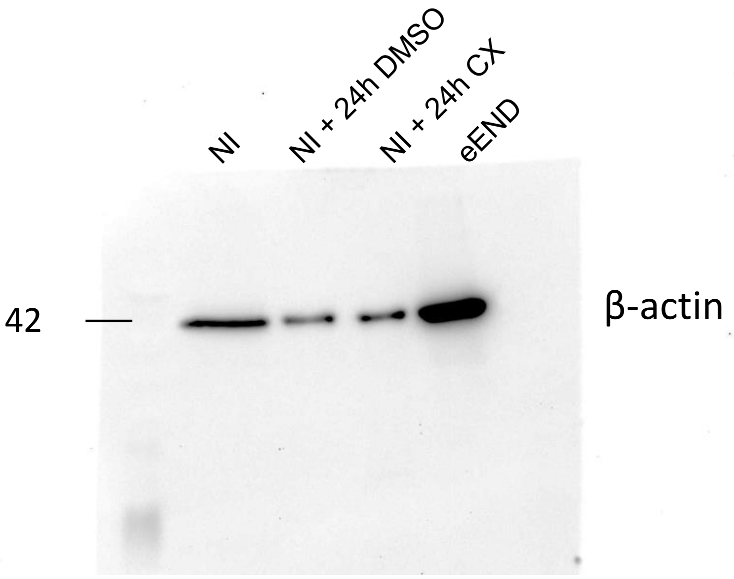

In the manuscript the middle two lanes are shown
NI: native islets, positive control for insulin
eEND: endothelial cells, negative control for insulin

Supplementary figure S1: Cell fractionation of glucose treated INS-1 cells

Figure S1 a, upper part of the blot (lower part used for anti USF2)
first antibody anti nucleolin

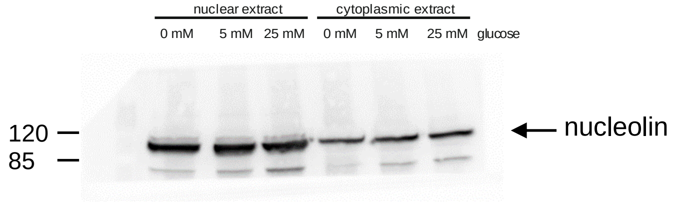

Figure S1 a, lower part of the blot
second antibody anti tubulin, same blot as for CK2a

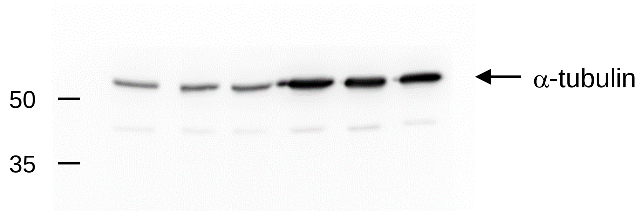

Figure S1 b, upper part of the blot
first antibody anti CK2a (1A5), same blot as for anti-tubulin

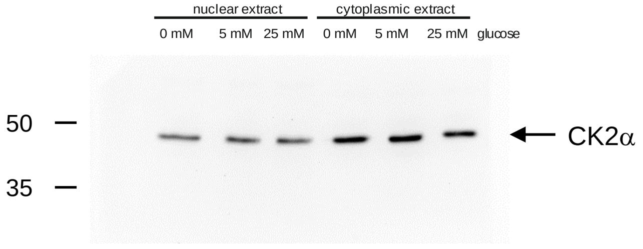

Figure S1 b, lower part of the blot (upper part used for anti USF1)
First antibody CK2b sc-46666

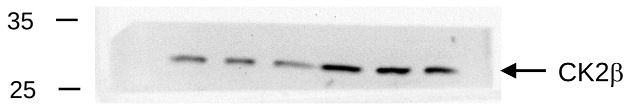

Supplementary figure S1: Cell fractionation of glucose treated INS-1 cells

Figure S1 c, upper part of the blot (lower part used for anti CK2β)
first antibody anti USF1 (sc-8983)

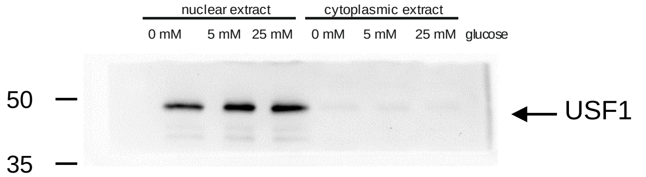

Figure S1 c, lower part of the blot (upper part used for nucleolin)
antibody anti USF2

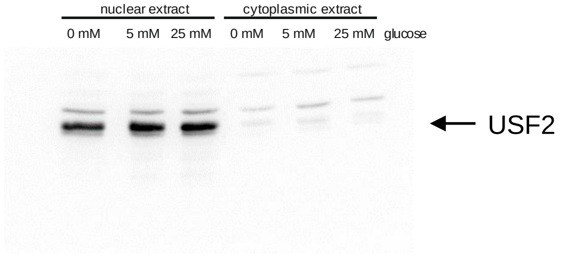

Figure S1 c, lower part of the blot
first antibody anti PDX-1

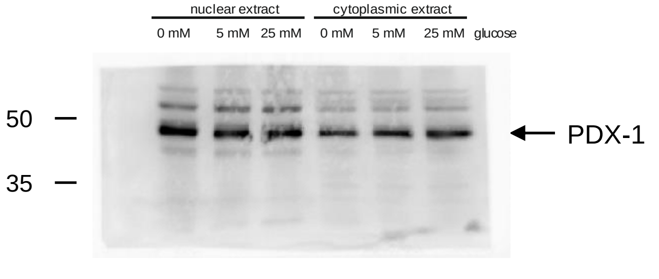

Supplement: Supplementary file 1 — Supplementary figure S1 [file 41598_2017_16590_MOESM1_ESM.pdf]
